# Cyclization of 5-hexynoic acid to 3-alkoxy-2-cyclohexenones

**DOI:** 10.3762/bjoc.7.155

**Published:** 2011-09-23

**Authors:** Anne T Hylden, Eric J Uzelac, Zeljko Ostojic, Ting-Ting Wu, Keely L Sacry, Krista L Sacry, Lin Xi, T Nicholas Jones

**Affiliations:** 1 Department of Chemistry, College of St. Benedict, St. John’s University, St. Joseph, MN 56374, USA

**Keywords:** acyl chlorides, alcohols, alkynes, cyclization, Lewis acids

## Abstract

The one-pot cyclization of 5-hexynoic acid to produce 3-alkoxy-2-cyclohexenones proceeds in good yields (58–90%). 3-Hexynoic acid was converted to its acyl chloride with the aid of oxalyl chloride and was cyclized to 3-chloro-2-cyclohexenone upon addition of indium(III) chloride. Subsequent addition of alcohol nucleophiles led to the desired 3-alkoxy-2-cyclohexenones.

## Findings

**Scheme 1:**
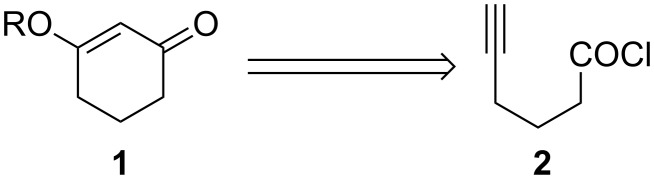
3-Alkoxy-2-cyclohexenones from the cyclization of 5-hexynoyl chloride.

Synthetic applications of 3-alkoxy-2-cyclohexenones toward the pursuit of natural products have been well documented [1–4]. Traditionally, 3-alkoxy-2-cyclohexenones have been prepared from 1,3-cyclohexadiones under a variety of conditions [5–6]. We report herein the cyclization of 5-hexynoyl chloride (**2**) as a complementary approach to the synthesis of 3-alkoxy-2-cyclohexenones **1** (Scheme 1).

The carbocyclization of 5-hexynoic acid was first reported by Tedder in 1957 [7]. Treatment of 5-hexynoic acid with trifluoroacetic anhydride followed by decomposition of the reaction mixture in methanol under reflux yielded 1,3-cyclohexandione in 25% yield. Later, Smit and coworkers reported the closely related acyclic reaction between alkynes and acyl cations leading to β-diketones in 25–76% yield [8–9]. Interestingly, only reactions run in mildly nucleophilic solvents, such as nitromethane, yielded products. Smit proposed β-nitronic esters, which were hydrolyzed upon work-up, as important intermediates in this reaction pathway.

With the work of Tedder and Smit in mind, we began our study with the cyclization of the known 5-hexynoyl chloride (**2**) [10] under Lewis acid conditions and quenching of the reaction with ethanol (Table 1). Our first cyclization attempt utilized silver tetrafluoroborate, which was found to be ineffective at promoting the cyclization. Use of aluminum chloride led to cyclization, but in very modest yield (26%). Titanium(IV) chloride was also screened, however no cyclization was observed. A dramatic increase in yield (71%) was obtained with the aid of indium(III) chloride. Additional metal chloride salts have been screened under our cyclization conditions (Table 1, entries 5–8). Although these salts promoted the desired cyclization, they were not found to be as effective as indium(III) chloride.

**Table 1:** Cyclization of 5-hexynoyl chloride to 3-ethoxy-2-cyclohexenone under Lewis acid conditions.

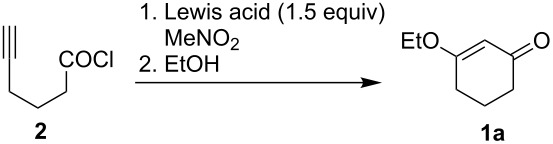		

Entry	Lewis acid	Yield

1	AgBF_4_	n.r.^a^
2	AlCl_3_	26%
3	TiCl_4_	n.r.^a^
4	InCl_3_	71%
5	BiCl_3_	17%
6	CuCl_2_	4%
7	ZnCl_2_	8%
8	FeCl_3_	63%

^a^n.r. = no reaction.

We did not observe formation of the alternative 5-membered cyclic product. This result is consistent with those reported by Smit whose acyclic cases led exclusively to acylation at the unsubstituted end of terminal alkynes [8]. Additionally, the preference for formation of the 6-membered cyclization product is likely biased by geometric factors due to the propylene tether linking the acyl chloride and alkyne moieties.

Recently we have developed one-pot conditions for this reaction starting from 5-hexynoic acid (**3**), see Table 2. ^1^H NMR analysis of aliquots from the reaction mixture indicated that **3** was completely converted to its acid chloride **2** within 2.5 hours. Indium(III) chloride was added directly to the flask upon formation of acid chloride **2**. Significantly, omitting nitromethane as a solvent did not hinder the reaction, indicating that the chloride ion concentration was sufficient to promote the cyclization event. In addition to simplifying the overall process, the yield was also improved from 71% to 81% for the ethyl case. Consistent by-products under these conditions were dialkyl oxalates formed from esterification of unreacted oxalyl chloride with excess alcohol nucleophiles, and thus a basic hydrolysis step was added to our work-up procedure. Secondary alcohols required heating to 90 °C following addition of the alcohol nucleophile to promote product formation. IR and NMR spectral data can be found in Supporting Information File 1.

**Table 2:** One-pot procedure for the cyclization of 5-hexynoyc acid to 3-alkoxy-2-cyclohexenones.

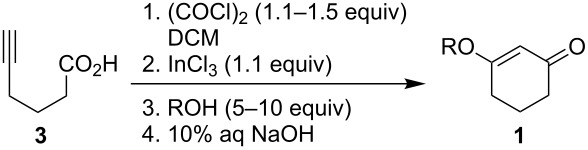		

Product	R	Yield

**1a**	Et	81%
**1b**	Me	72%
**1c**	*n*-Pr	89%
**1d**	iPr	74%^a^
**1e**	*n*-Bu	74%
**1f**	*s*-Bu	70%^a^
**1g**	*t*-Bu	0%^b^
**1h**	Ph	62%
**1i**	Bn	58%
**1j**	CH_2_=CH–CH_2_	90%

^a^Required heating to 90 °C following the addition of alcohol nucleophiles to promote product formation. ^b^Only 3-chloro-2-cyclohexenone was observed in the ^1^H NMR spectrum of the crude reaction mixture.

**Scheme 2:**
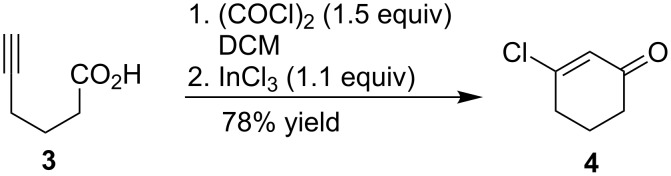
Preparation of 3-chloro-2-cyclohexenone (**4**).

Additional evidence for the participation of chloride ion in this reaction was given by the isolation of the known 3-chloro-2-cyclohexenone (**4**) [11] from reactions that involved sterically hindered alcohols such as *tert*-butanol (Table 2). Enone **4** was prepared directly in 78% yield under our cyclization conditions except that the addition of an alcohol nucleophile was omitted (Scheme 2).

**Scheme 3:**
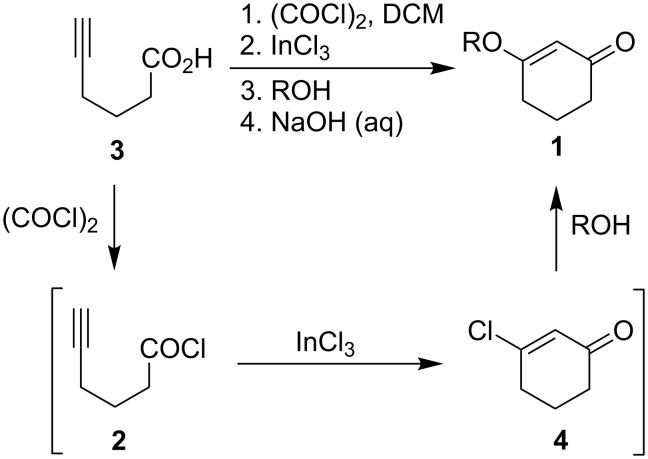
Proposed pathway for the one-pot conversion of 5-hexynoic acid to 3-alkoxy-2-cyclohexenones.

Based on the isolation of **4** and the apparent steric influence of the alcohol nucleophiles, as observed in the yield data in Table 2, we have proposed a possible pathway for this process (Scheme 3). Acid **3** was converted to the acid chloride **2** by treatment of oxalyl chloride [10]. In situ addition of indium(III) chloride promoted cyclization of **2** to 3-chloro-2-cyclohexenone (**4**) and was followed by quenching with alcohol nucleophiles, yielding 3-alkoxy-2-cyclohexenones **1**.

Related examples by Forsyth [2] and Clausen [4] support our proposed mechanism. They reported conjugate addition of alcohol nucleophiles to 3-chloro-2-cyclohexenone substrates under basic conditions followed by elimination of the chloride. The key difference between their reported conditions and ours is that our cyclizations are Lewis acid mediated.

Building on the work of Tedder [7] and Smit [8–9] we have improved the alkynoic acid cyclization approach in a manner that allows ready access to 3-alkoxy-2-cyclohexenones in good yields. Further elaboration of this methodology is ongoing.

## Experimental

**General methods**: All experiments were carried out under an atmosphere of dry nitrogen in oven-dried glassware. Solvents, starting materials and reagents were purchased (Sigma-Aldrich and Fisher Scientific) and used without further purification. ^1^H and ^13^C NMR spectra were obtained on a Varian Gemini 2000 (300 MHz) spectrometer with CDCl_3_ as solvent. Infrared spectra were obtained on a Thermo Nicolet 380 FT-IR spectrometer with Smart IR ATR (Si or ZnSe). Mass spectra were taken with a Varian 3800 GC/Saturn 2000 MS (ion trap).

**3-Methoxy-2-cyclohexenone (1b):** 5-Hexynoic acid (**3**) (0.267 g, 2.38 mmol) was combined with oxalyl chloride (0.396 g, 3.05 mmol) in anhydrous dichloromethane (1.5 mL) in a nitrogen-purged 25 mL round-bottom flask and stirred for 2.5 hours. Indium(III) chloride (0.565 g, 2.55 mmol) was added and the mixture was stirred for 3 hours. Methanol (0.401 g, 12.5 mmol) was then added and the mixture was stirred for 15 minutes. Next, a saturated NaHCO_3_ solution (6 mL) and THF (3 mL) were added and stirred for 15 minutes, followed by addition of NaOH (10% aq, 6 mL) and 5 minutes of stirring. The mixture was extracted with dichloromethane (3 × 10 mL) and the combined organic layers were dried over CaCl_2_ and concentrated in vacuo. Solvent exchange with CHCl_3_ (2 × 5 mL) gave the product (0.216 g, 1.71 mmol, 72%) as an oil. ^1^H NMR (300 MHz, CDCl_3_) δ 5.36 (s, 1H, CH), 3.68 (s, 3H, CH_3_), 2.40 (t, *J* = 6.3 Hz, 2H, CH_2_), 2.34 (t, *J* = 6.5 Hz, 2H, CH_2_), 1.97 (tt, *J* = 6.3, 6.5 Hz, 2H, CH_2_); ^13^C NMR (300 MHz, CDCl_3_) δ 195.1, 174.3, 97.6, 50.9, 31.9, 24.0, 16.4; IR (ATR, neat) 2946, 1645, 1603, 1378, 1225, 1186, 1135, 1003, 826, 757 cm^−1^; EIMS (*m/z*): 127.

**3-Isopropoxy-2-cyclohexenone (1d)**: 5-Hexynoic acid (0.241 g, 2.15 mmol) and oxalyl chloride (0.361 g, 2.84 mmol) were combined with anhydrous dichloromethane (1.5 mL) in a nitrogen-purged 25 mL round-bottom flask and stirred for 2.5 hours. Indium(III) chloride (0.522 g, 2.36 mmol) was added and the mixture was stirred for 3 hours. 2-propanol (1.352 g, 22.5 mmol) was then added and the mixture was stirred for 15 minutes at 90 °C. The reaction mixture was cooled to room temperature and a saturated NaHCO_3_ solution (6 mL) and THF (3 mL) were added and stirred for 15 minutes, followed by addition of NaOH (10% aq, 6 mL) and 15 more minutes of stirring. The mixture was extracted with DCM (2 × 15 mL) and the combined organic layers were dried over CaCl_2_ and concentrated in vacuo. Solvent exchange with CHCl_3_ (3 × 5 mL) gave the pure product (0.245 g, 1.59 mmol, 74%) as an oil. ^1^H NMR (300 MHz, CDCl_3_) δ 5.33 (s, 1H, CH), 4.41 (sept., *J* = 6.1 Hz, 1H, CH), 2.35 (t, *J* = 6.5 Hz, 2H, CH_2_), 2.32 (t, *J* = 7.1 Hz, 2H, CH_2_), 1.95 (tt, *J* = 6.5, 7.1 Hz, 2H, CH_2_), 1.27 (d, *J* = 6.1 Hz, 6H, 2 CH_3_); ^13^C NMR (CDCl_3_) δ 195.4, 172.4, 98.3, 66.2, 31.9, 24.8, 16.6; IR (ATR, neat): 2980, 1645, 1594, 1379, 1219, 1183, 1105, 940, 824, 752 cm^−1^; EIMS (*m/z*): 155.

### ^1^H NMR data for compounds **1a**, **1c**, **1e**, **1f**, **1h**–**j** and **4**

**3-Ethoxy-2-cyclohexenone (1a)**: ^1^H NMR (300 MHz, CDCl_3_) δ 5.34 (s, 1H, CH), 3.89 (q, *J* = 7.1 Hz, 2H, CH_2_), 2.39 (t, *J* = 6.3 Hz, 2H, CH_2_), 2.34 (t, *J* = 6.5 Hz, 2H, CH_2_), 1.97 (tt, *J* = 6.3, 6.5 Hz, 2H, CH_2_), 1.35 (t, *J* = 7.1 Hz, 3H, CH_3_).

**3-Propoxy-2-cyclohexenone (1c)**: ^1^H NMR (300 MHz, CDCl_3_) δ 5.33 (s, 1H, CH), 3.76 (t, *J* = 6.5 Hz, 2H, CH_2_), 2.39 (t, *J* = 6.3 Hz, 2H, CH_2_), 2.32 (t, *J* = 6.5 Hz, 2H, CH_2_), 1.96 (tt, *J* = 6.3, 6.5 Hz, 2H, CH_2_), 1.73 (tq, *J* = 6.5, 7.1 Hz, 2H, CH_2_), 0.96 (t, *J* = 7.4 Hz, 2H, CH_3_).

**3-Butoxy-2-cyclohexenone (1e)**: ^1^H NMR (300 MHz, CDCl_3_) δ 5.33 (s, 1H, CH), 3.81 (t, *J* = 6.5 Hz, 2H, CH_2_), 2.38 (t, *J* = 6.3 Hz, 2H, CH_2_), 2.32 (t, *J* = 6.6 Hz, 2H, CH_2_), 1.96 (tt, *J* = 6.3, 6.6 Hz, 2H, CH_2_), 1.69 (tt, *J* = 6.5, 7.4 Hz, 2H, CH_2_), 1.41 (tq, *J* = 7.4 Hz, 2H, CH_2_), 0.93 (t, *J* = 7.4 Hz, 2H, CH_2_).

**3-(*sec*-Butoxy)-2-cyclohexenone (1f)**: ^1^H NMR (300 MHz, CDCl_3_) δ 5.32 (s, 1H, CH), 4.19 (m, 1H, CH), 2.36 (t, *J* = 6.3 Hz, 2H, CH_2_), 2.33 (t, *J* = 6.6 Hz, 2H, CH_2_), 1.96 (tt, *J* = 6.3, 6.6 Hz, 2H, CH_2_), 1.61 (m, *2*H, CH_2_), 1.24 (d, *J* = 6.2 Hz, 3H, CH_3_), 0.90 (t, *J* = 7.5 Hz, 3H, CH_3_).

**3-Phenoxy-2-cyclohexenone (1h)**: ^1^H NMR (300 MHz, CDCl_3_) δ 7.37 (t, *J* = 7.6 Hz, 2H, 2 CH), 7.22 (dd, *J* = 7.6, 7.7 Hz, 1H, CH), 7.02 (d, *J* = 7.7 Hz, 2H, 2 CH), 5.10 (s, 1H, CH), 2.64 (t, *J* = 6.3 Hz, 2H, CH_2_), 2.36 (t, *J* = 6.5 Hz, 2H, CH_2_), 2.06 (tt, *J* = 6.3, 6.5 Hz, 2H, CH_2_).

**3-Benzyloxy-2-cyclohexenone (1i)**: ^1^H NMR (300 MHz, CDCl_3_) δ 7.37 (m, 5H, 5 CH), 5.48 (s, 1H, CH), 4.89 (s, 2H, CH_2_), 2.47 (t, *J* = 6.3 Hz, 2H, CH_2_), 2.37 (t, *J* = 6.5 Hz, 2H, CH_2_), 2.00 (tt, *J* = 6.3, 6.5 Hz, 2H, CH_2_).

**3-Allyloxy-2-cyclohexenone (1j)**: ^1^H NMR (300 MHz, CDCl_3_) δ 5.95 (ddt, *J* = 5.6, 9.2, 15.4 Hz, 1H, CH), 5.38 (dd, *J* = 1.4, 9.2 Hz, 1H, CH), 5.35 (s, 1H, CH), 5.29 (dd, *J* = 1.4, 5.6 Hz, 1H, CH), 4.36 (d, *J* = 5.6 Hz, 2H, CH_2_), 2.42 (t, *J* = 6.2 Hz, 2H, CH_2_), 2.33 (t, *J* = 6.6 Hz, 2H, CH_2_), 1.97 (tt, *J* = 6.2, 6.6 Hz, 2H, CH_2_).

**3-Chloro-2-cyclohexenone (4)**: ^1^H NMR (300 MHz, CDCl_3_) δ 6.21 (s, 1H, CH), 2.68 (t, *J* = 6.1 Hz, 2H, CH_2_), 2.39 (t, *J* = 6.7 Hz, 2H, CH_2_), 2.08 (tt, *J* = 6.1, 6.7 Hz, 2H, CH_2_).

## Supporting Information

File 1 IR and NMR data for compounds **1a–f**, **1h–j** and **4**.
