# Supplementary material for: Cyclization of 5-hexynoic acid to 3-alkoxy-2-cyclohexenones
Source: Beilstein J Org Chem. 2011 Sep 23;7:1323–6. doi: 10.3762/bjoc.7.155 (PMC3201045; doi:10.3762/bjoc.7.155)

Supporting Information

for

Cyclization of 5-hexynoic acid to 3-alkoxy-2-cyclohexenones

Anne T. Hylden, Eric J. Uzelac, Zeljko Ostojic, Ting-Ting Wu, Keely L. Sacry, Krista L. Sacry, Lin Xi and T. Nicholas Jones*

Address: Department of Chemistry, College of St. Benedict, St. John's University, St. Joseph, MN 56374, USA

Email: T. Nicholas Jones - tjones@csbsju.edu

* Corresponding author

IR and NMR data for compounds **1a–f, 1h–j** and **4**

pp S1–S31

Compound 1a – ^{1}H NMR

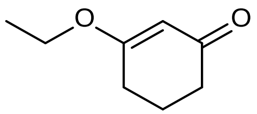

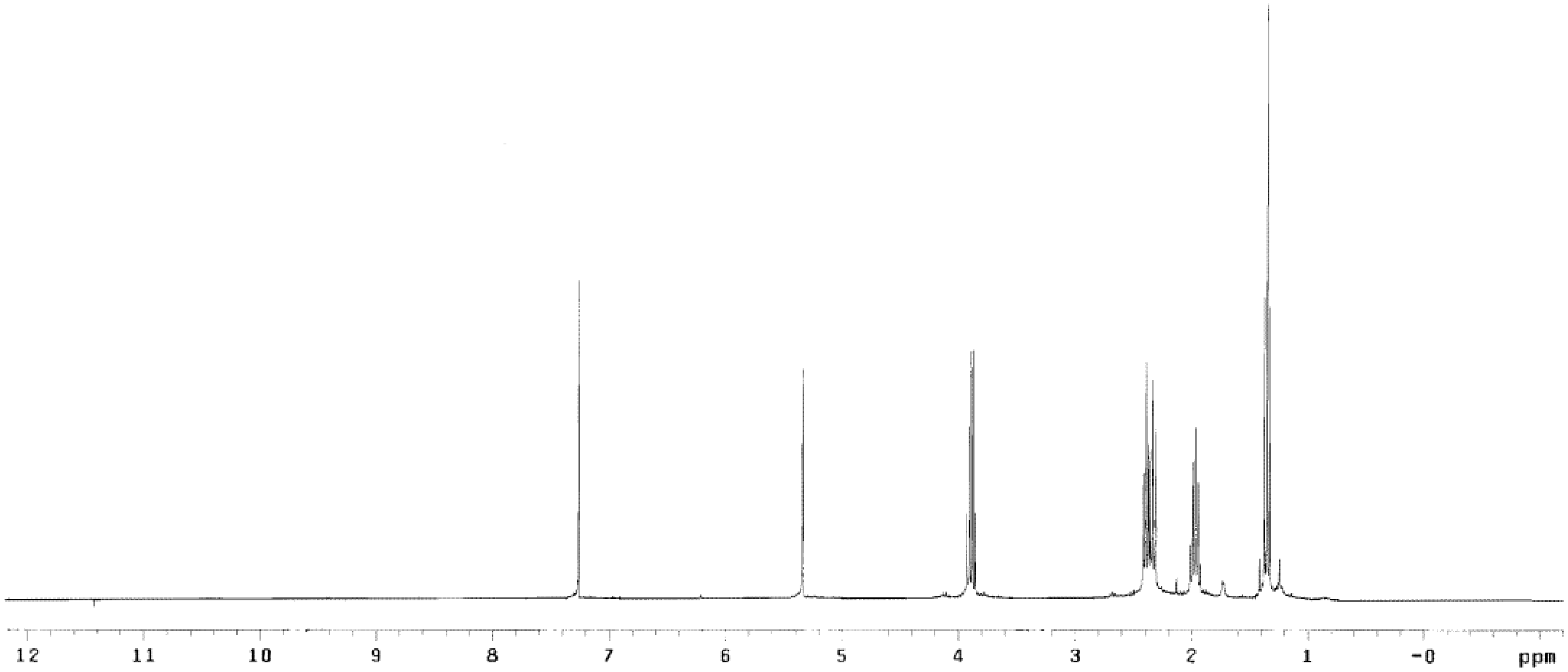

Compound 1a – ^{13}C NMR

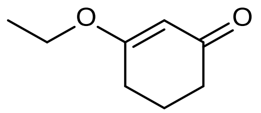

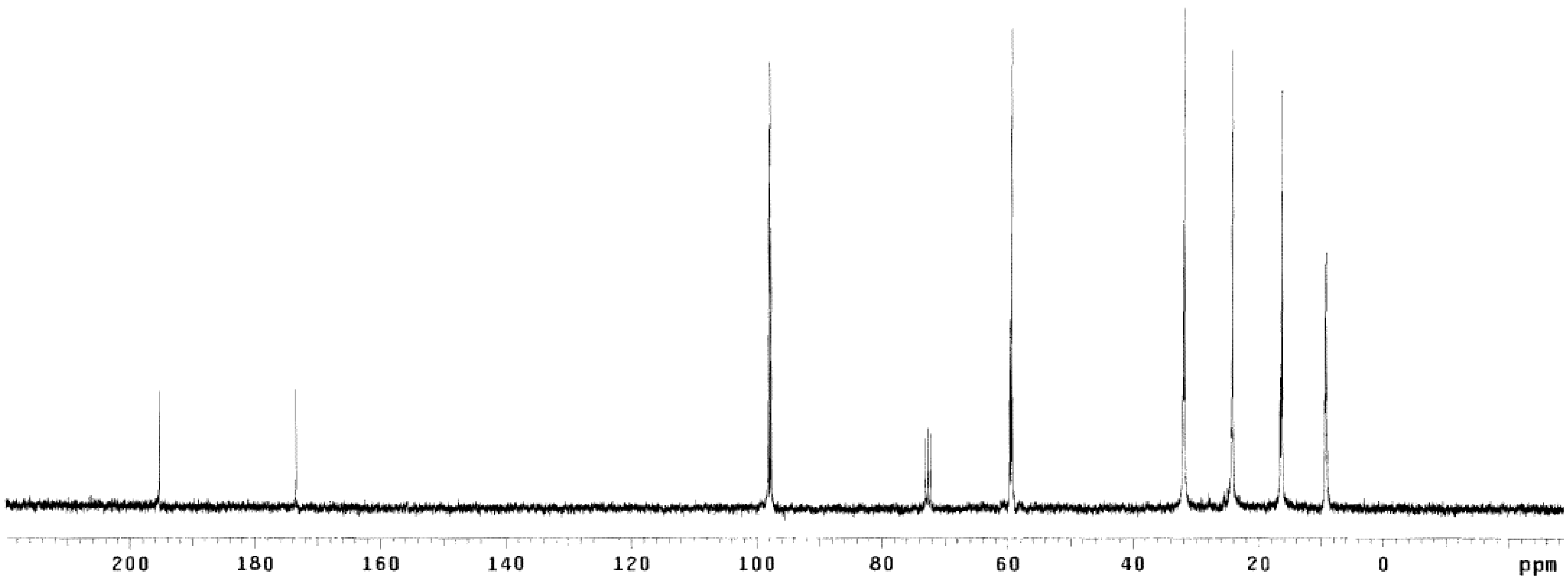

Compound 1a – IR

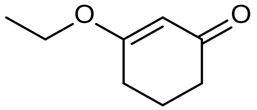

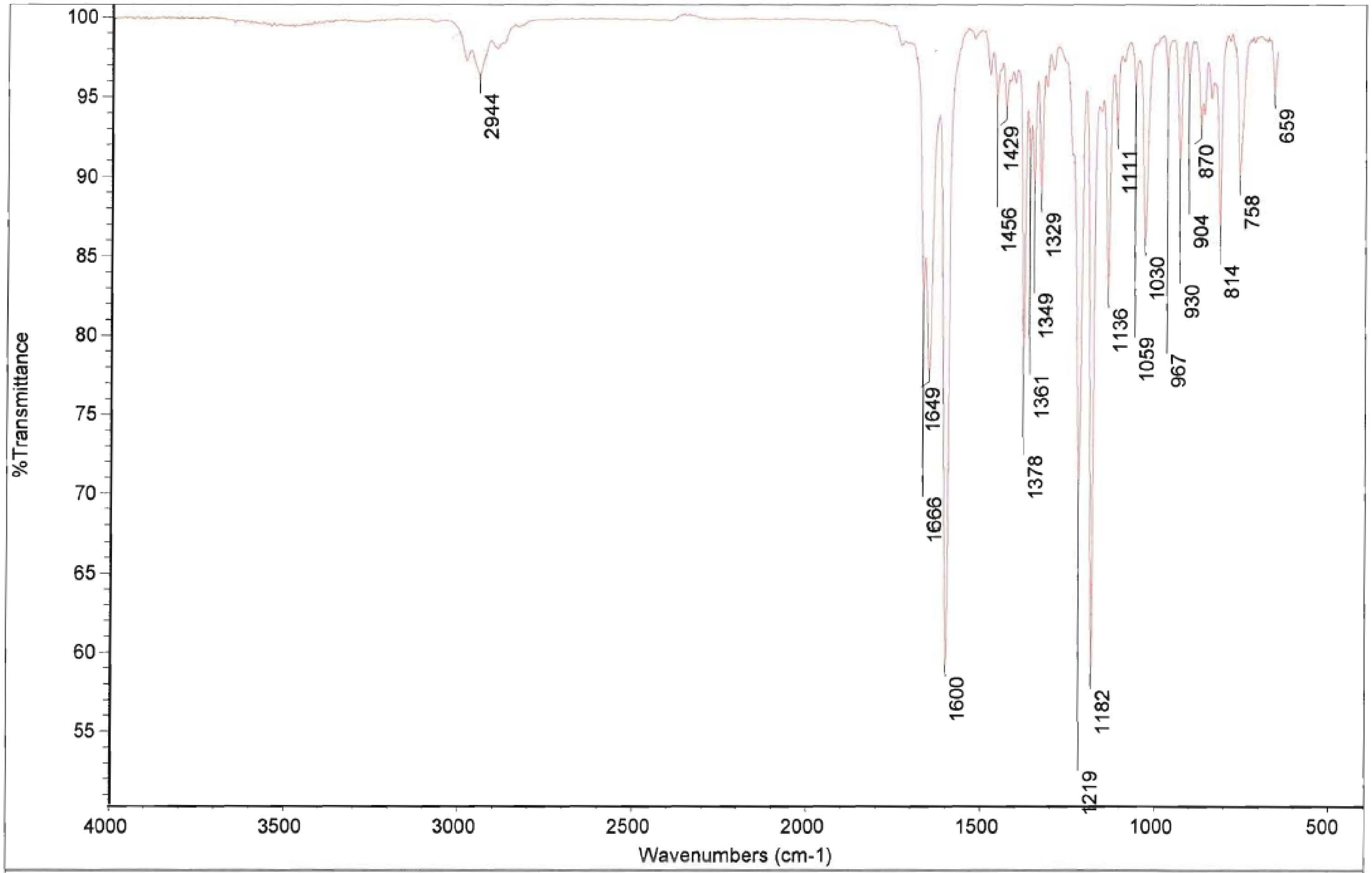

Date: Fri Jul 17 13:07:29 2009 (GMT-05:00) ath-ethoxy-7/17/09

Scans: 16

Resolution: 4.000

Compound 1b – ^{1}H NMR

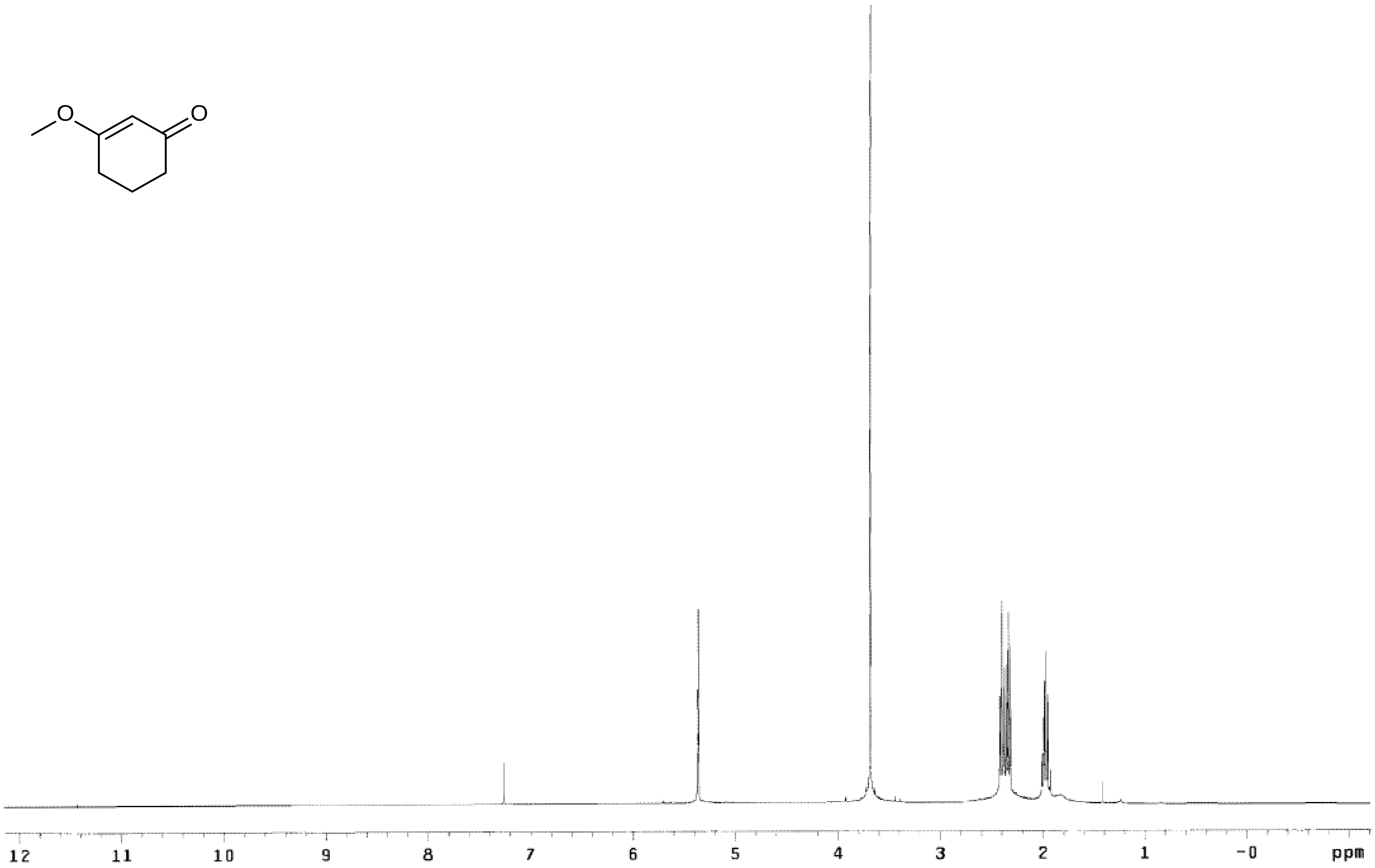

Compound 1b – ^{13}C NMR

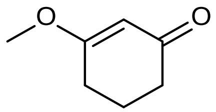

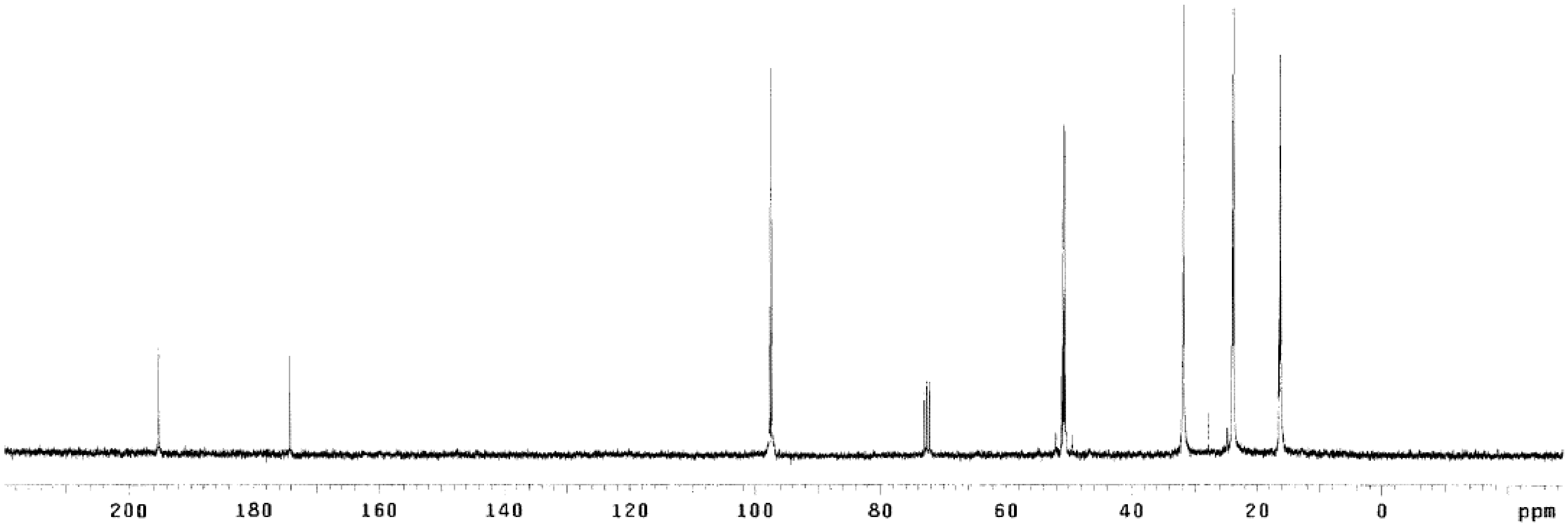

Compound 1b – IR

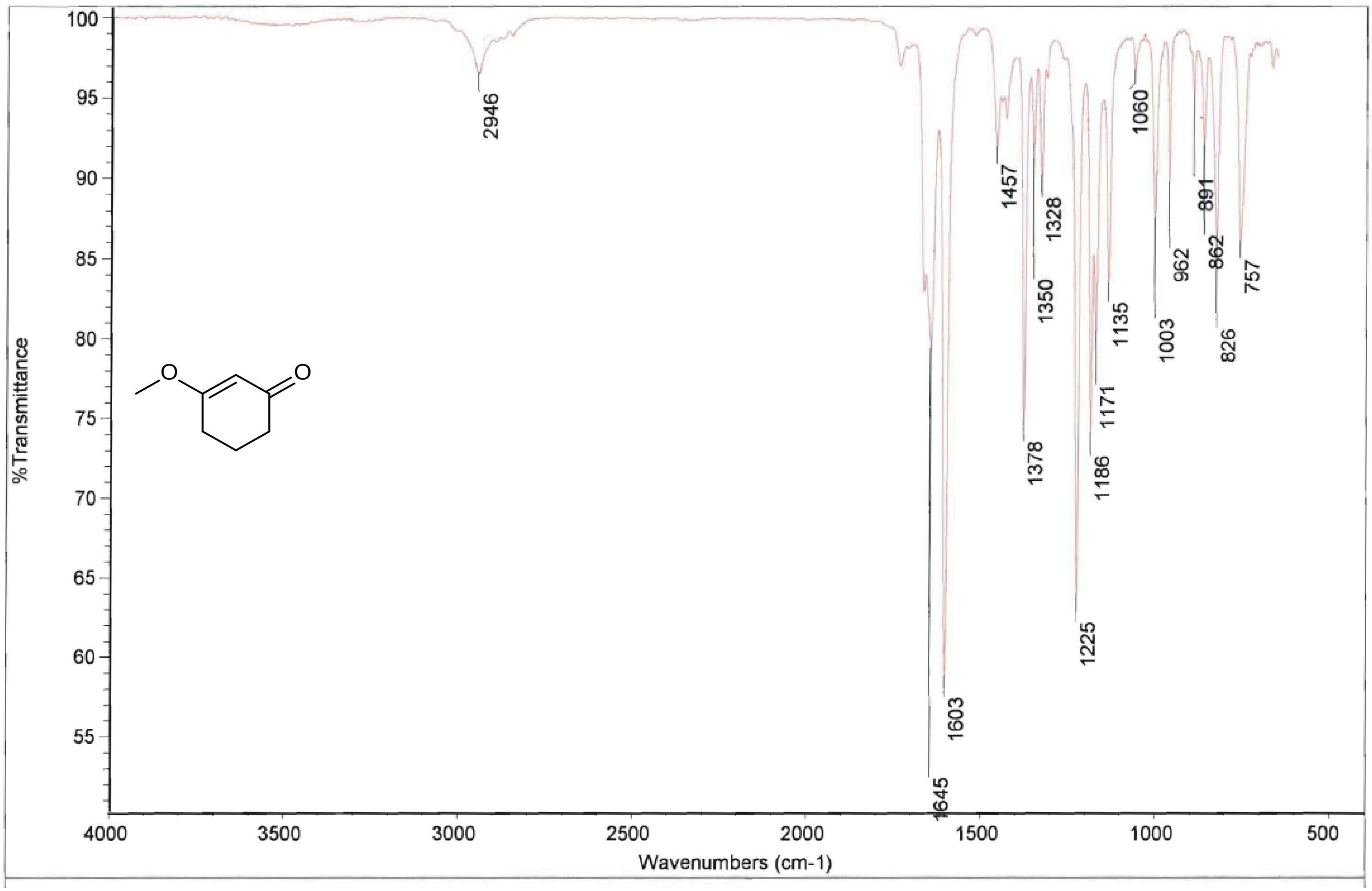

Compound 1c – ^{1}H NMR

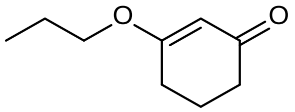

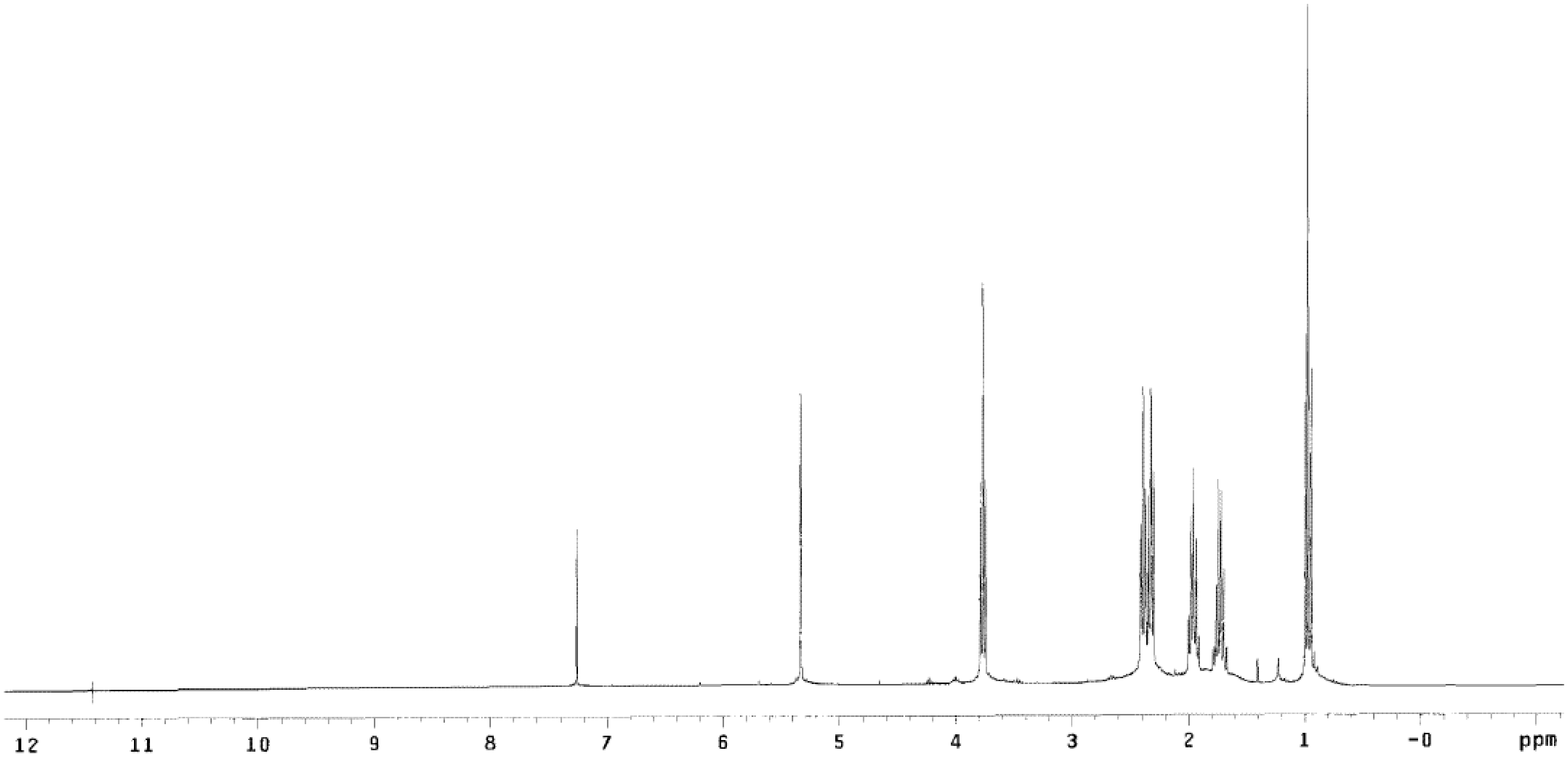

Compound 1c – ^{13}C NMR

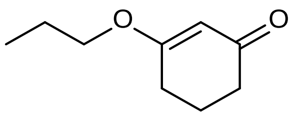

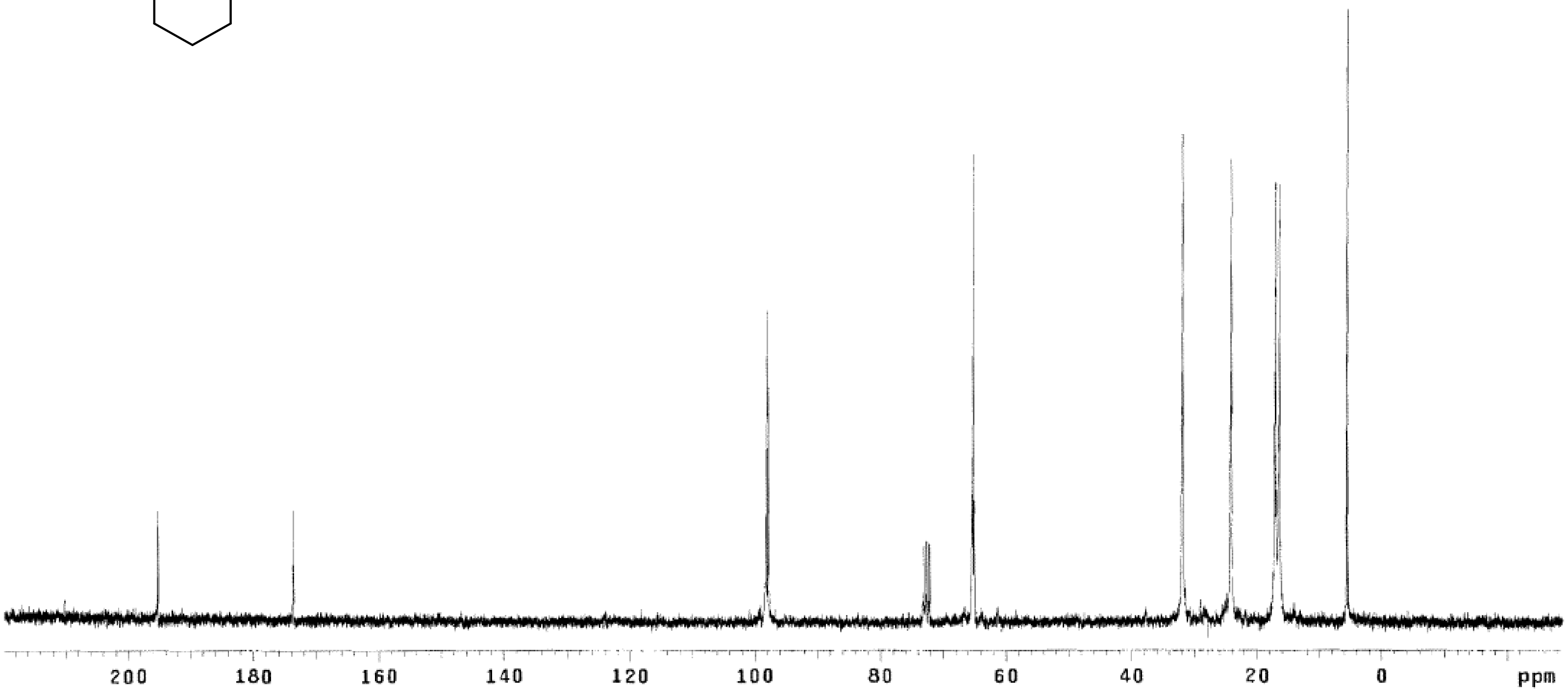

Compound 1c – IR

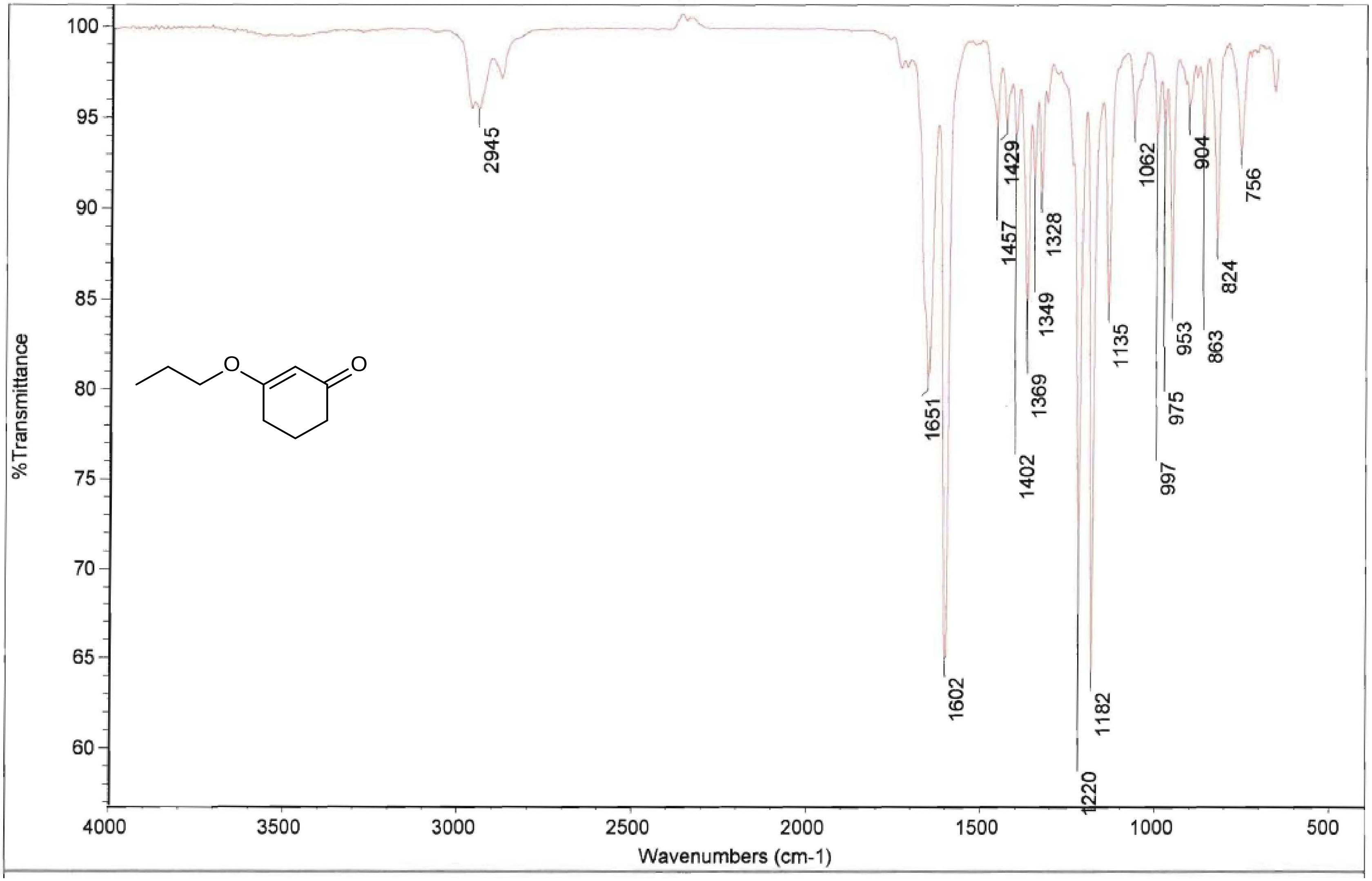

Compound 1d – ^{1}H NMR

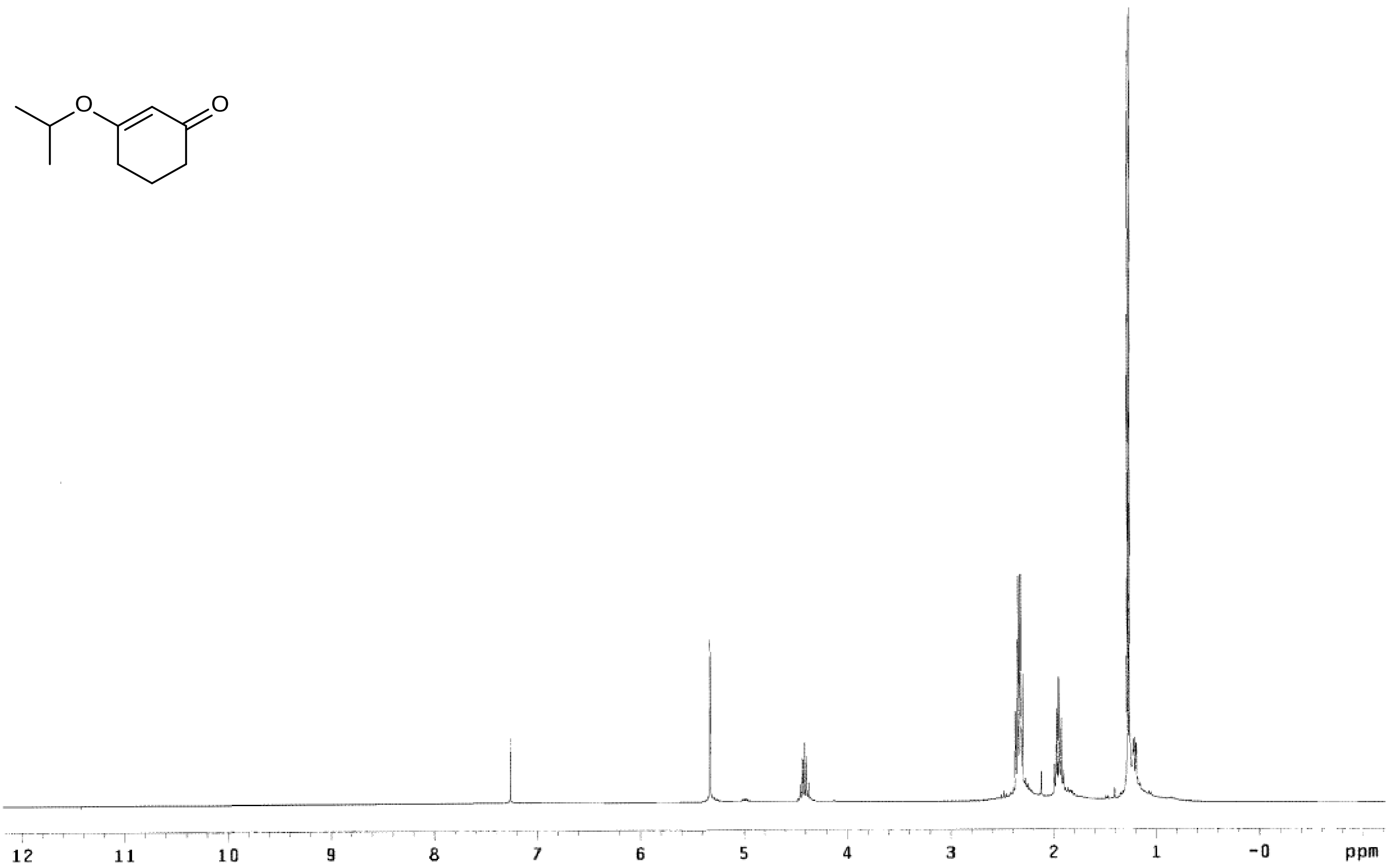

Compound 1d – ^{13}C NMR

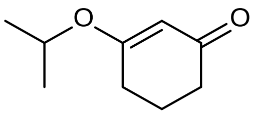

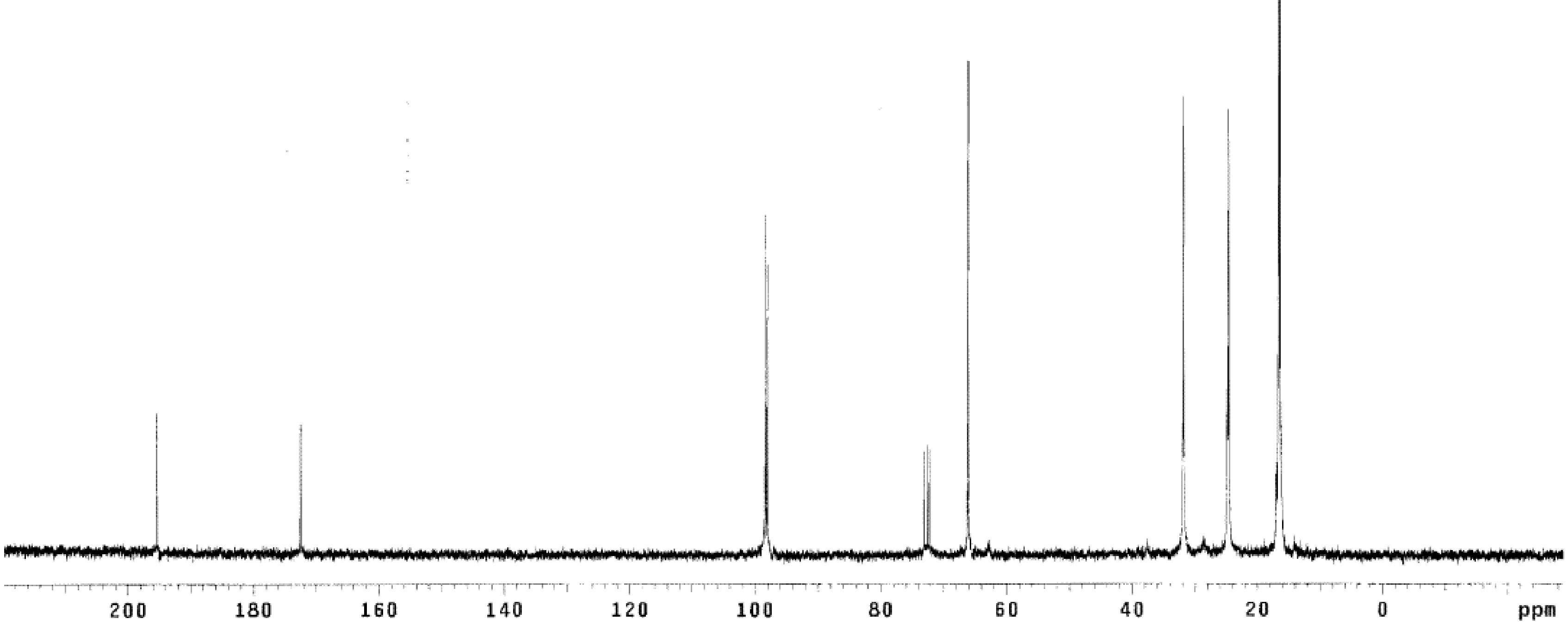

Compound 1d – IR

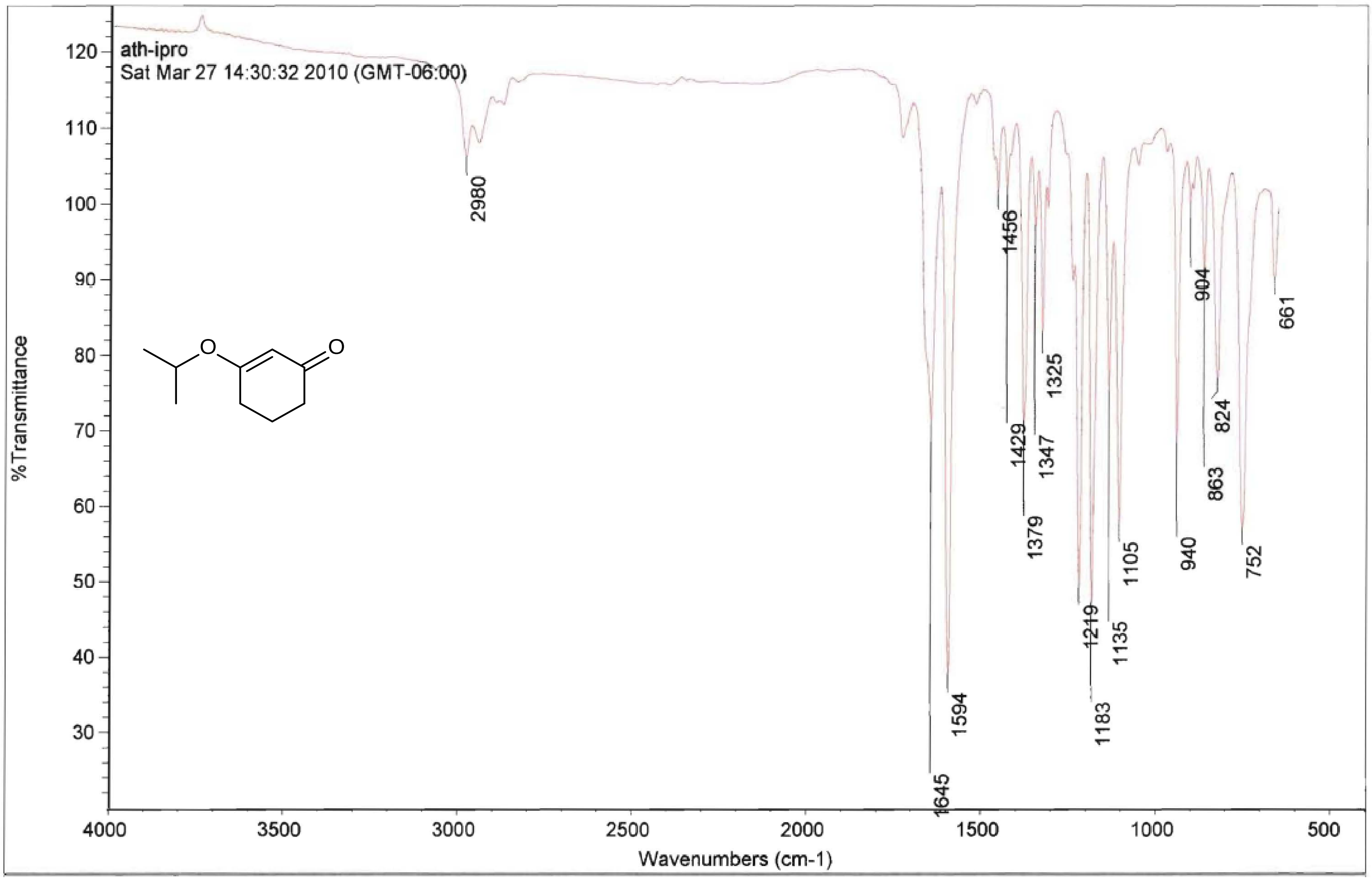

Compound 1e – ^{1}H NMR

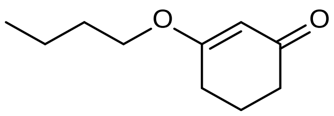

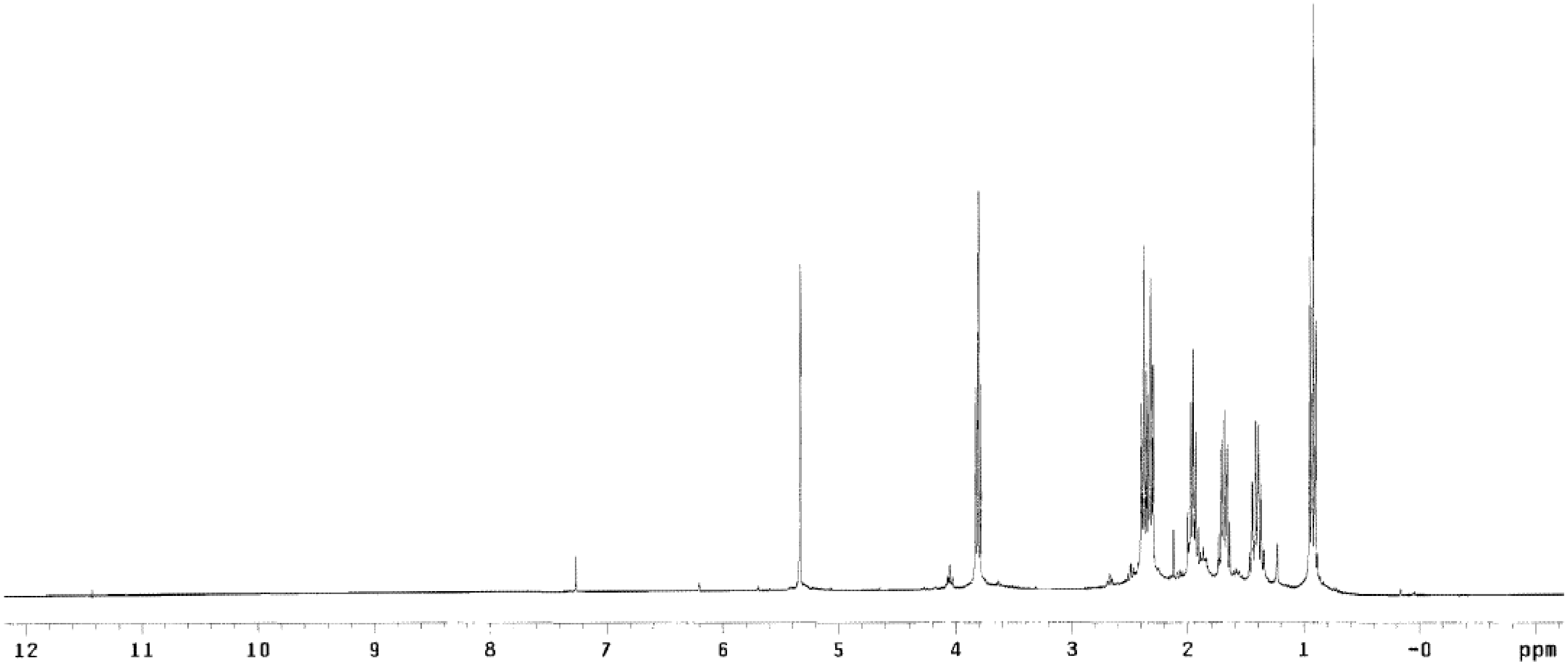

Compound 1e – ^{13}C NMR

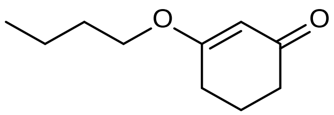

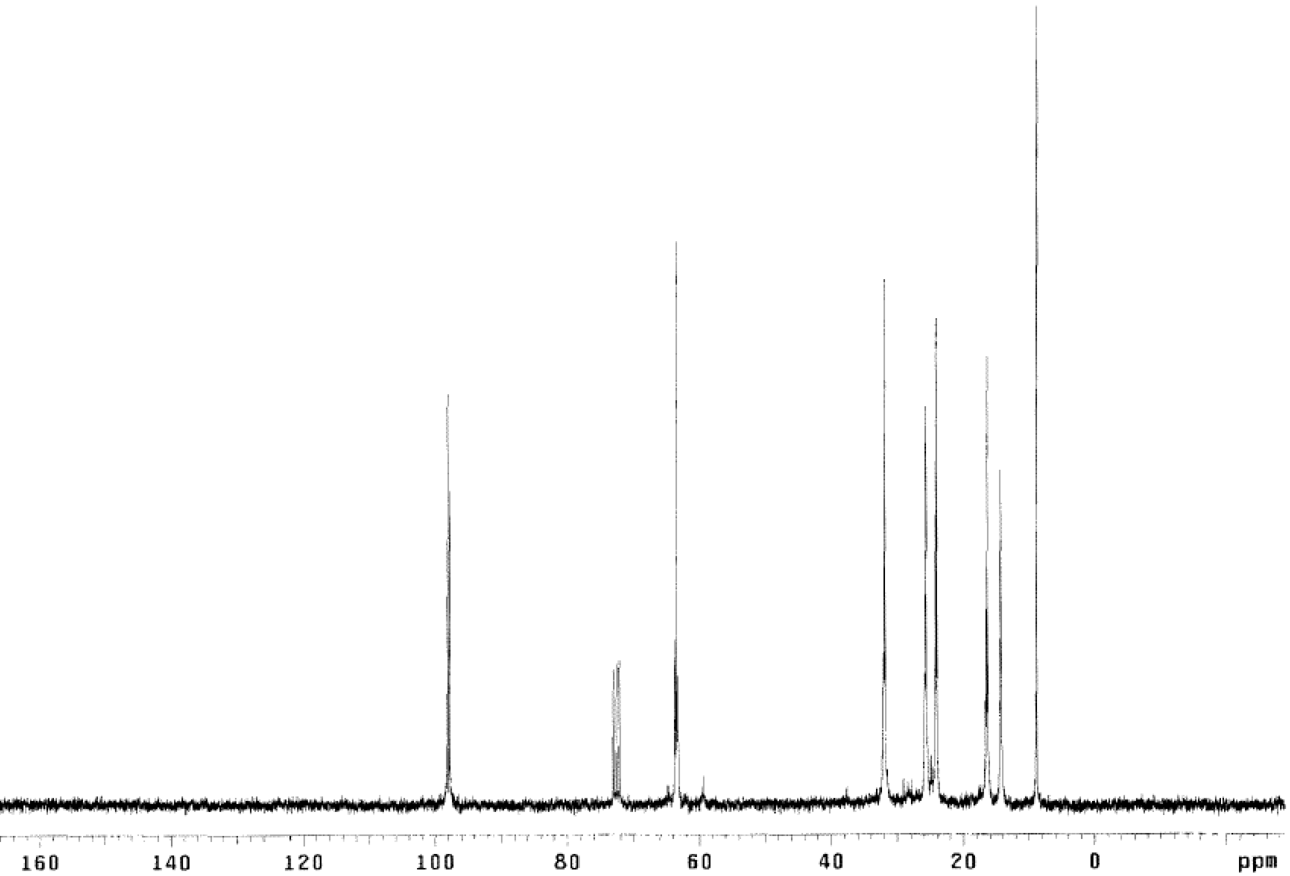

Compound 1e – IR

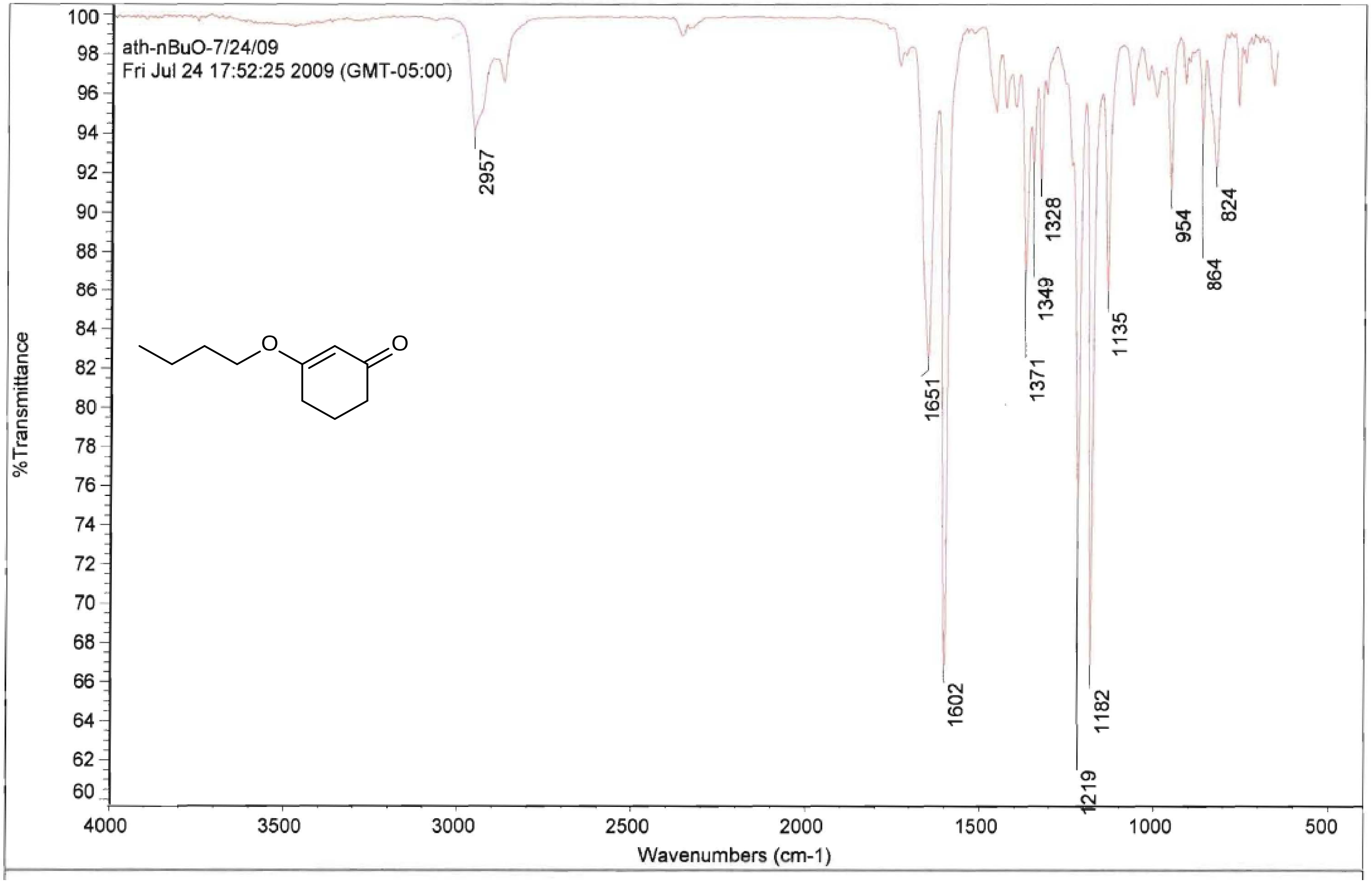

Compound 1f – ^{1}H NMR

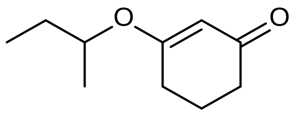

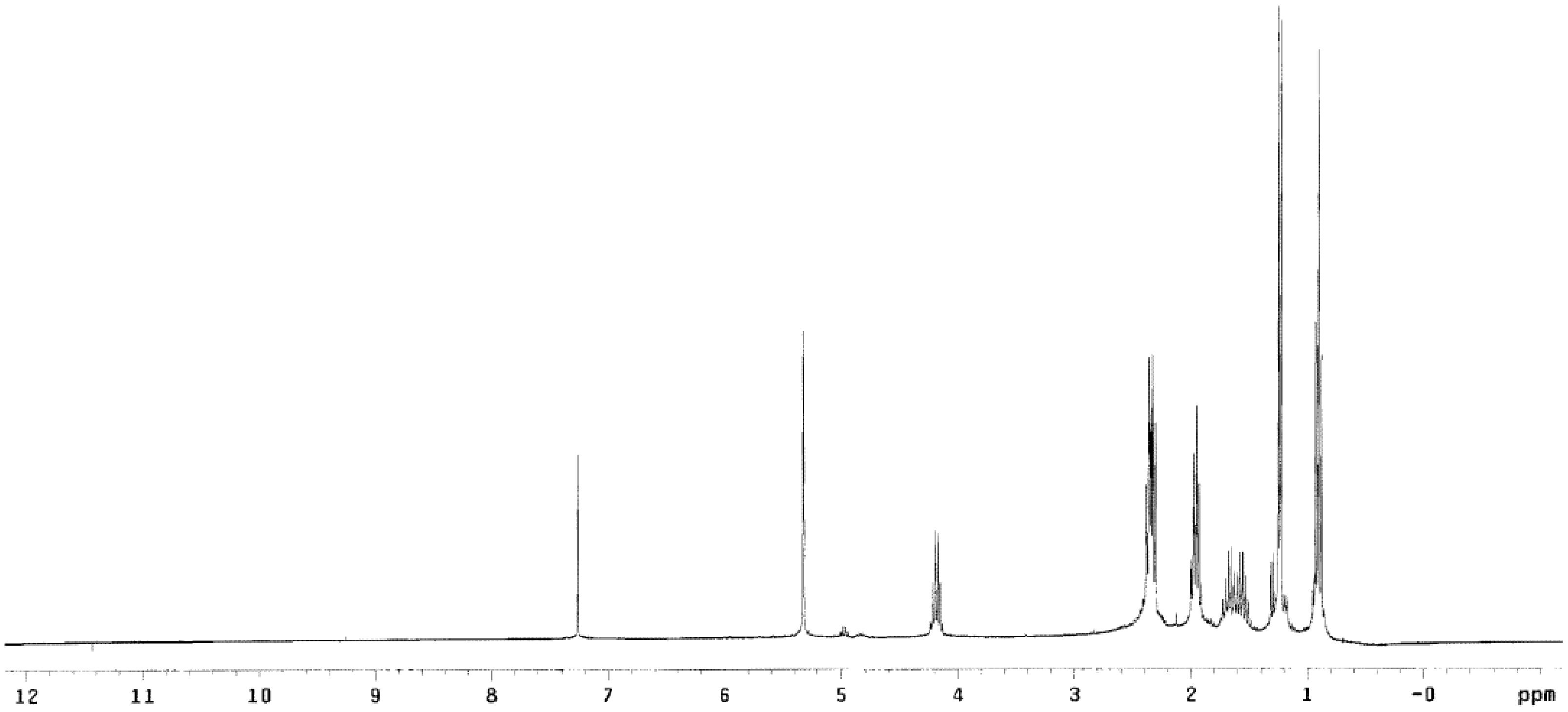

Compound 1f – ^{13}C NMR

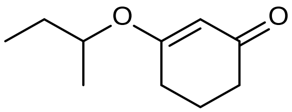

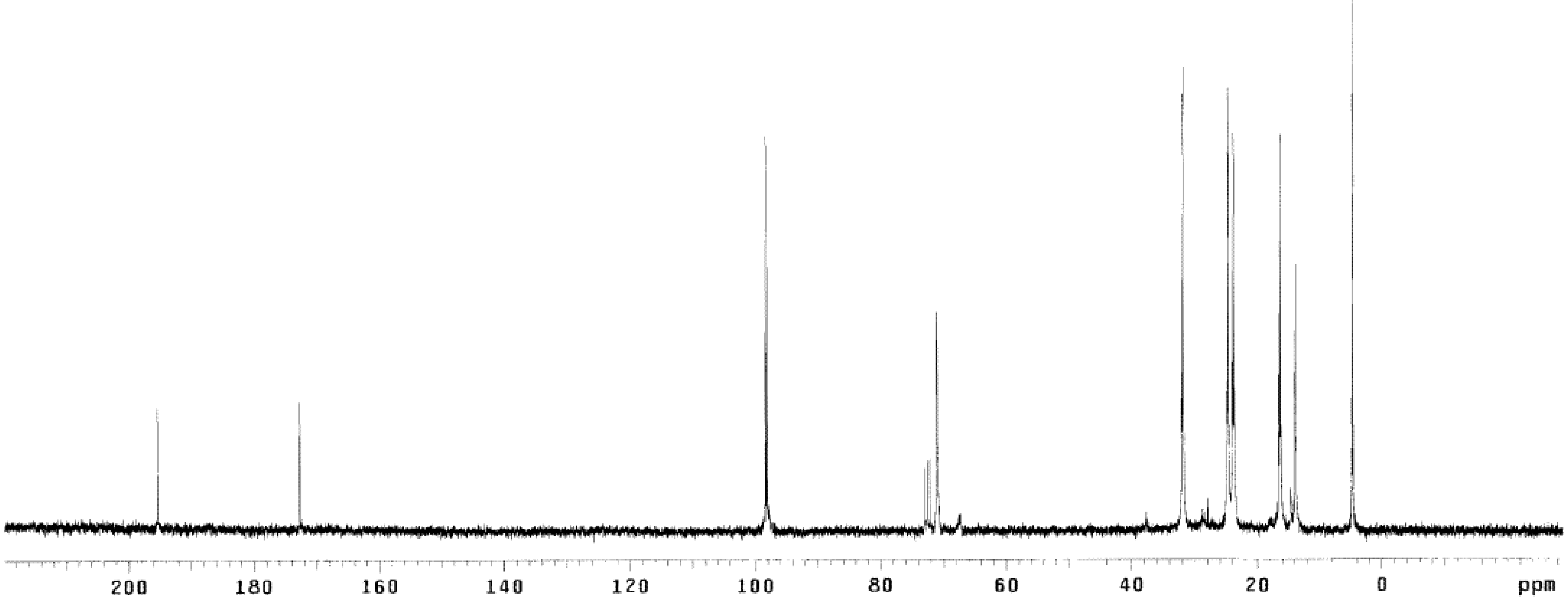

Compound 1f – IR

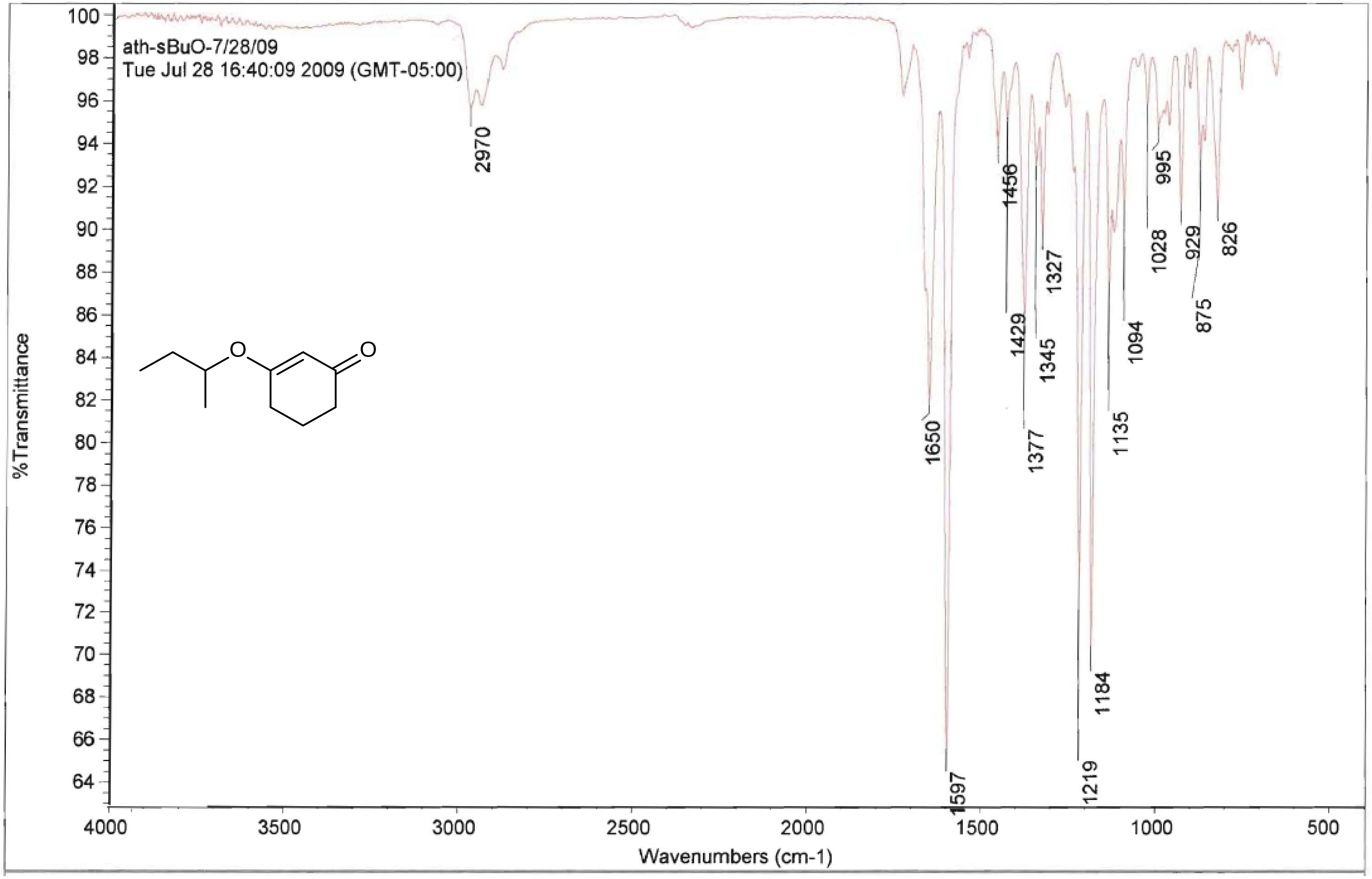

Compound 1h – ^{1}H NMR

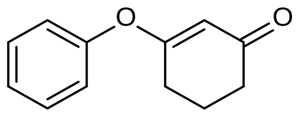

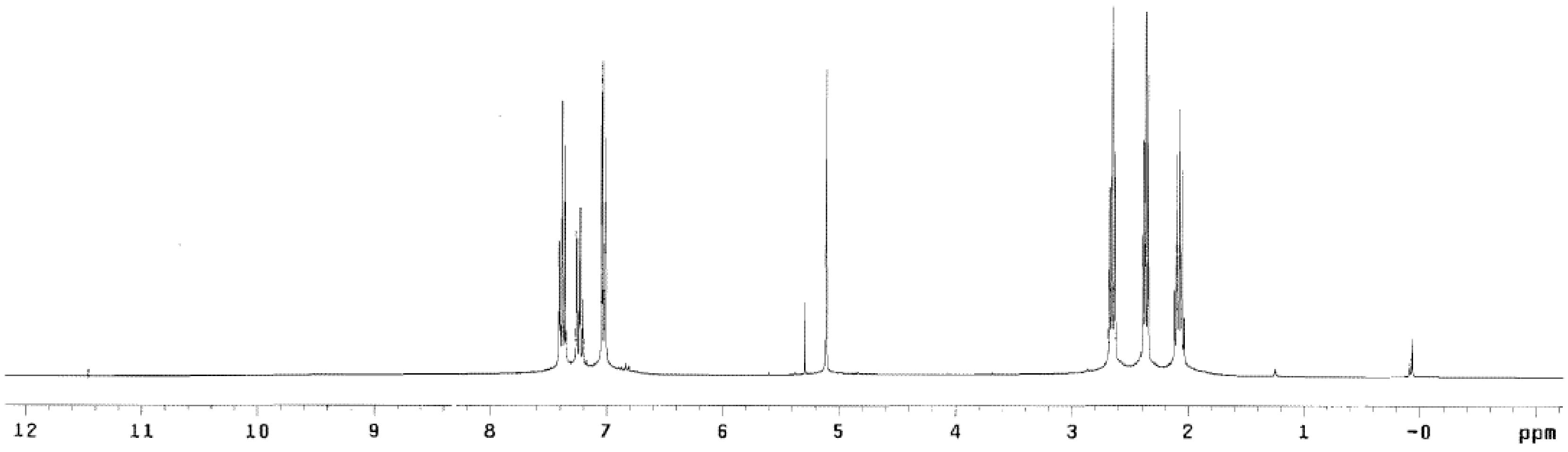

Compound 1h – ^{13}C NMR

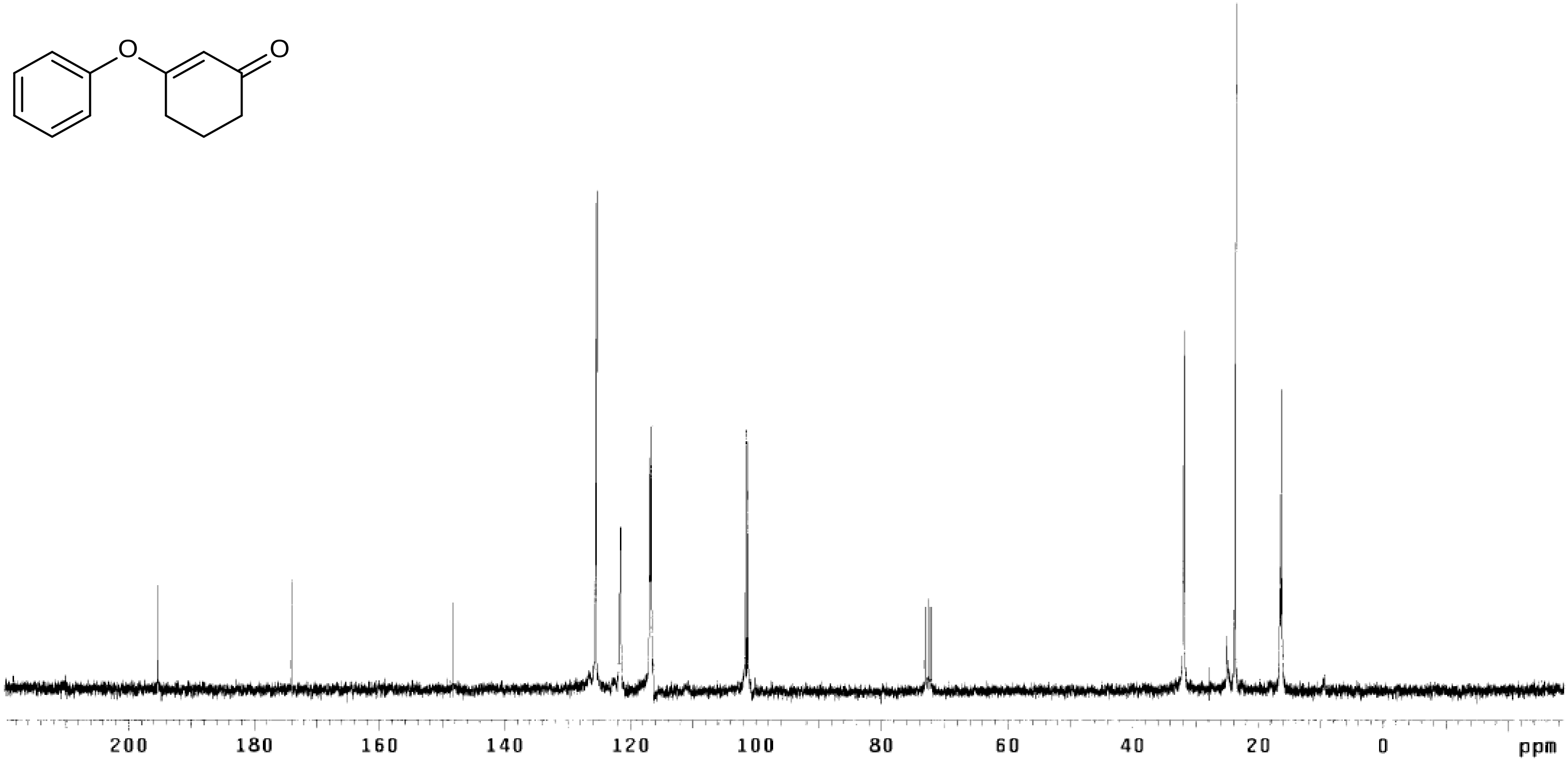

Compound 1h – IR

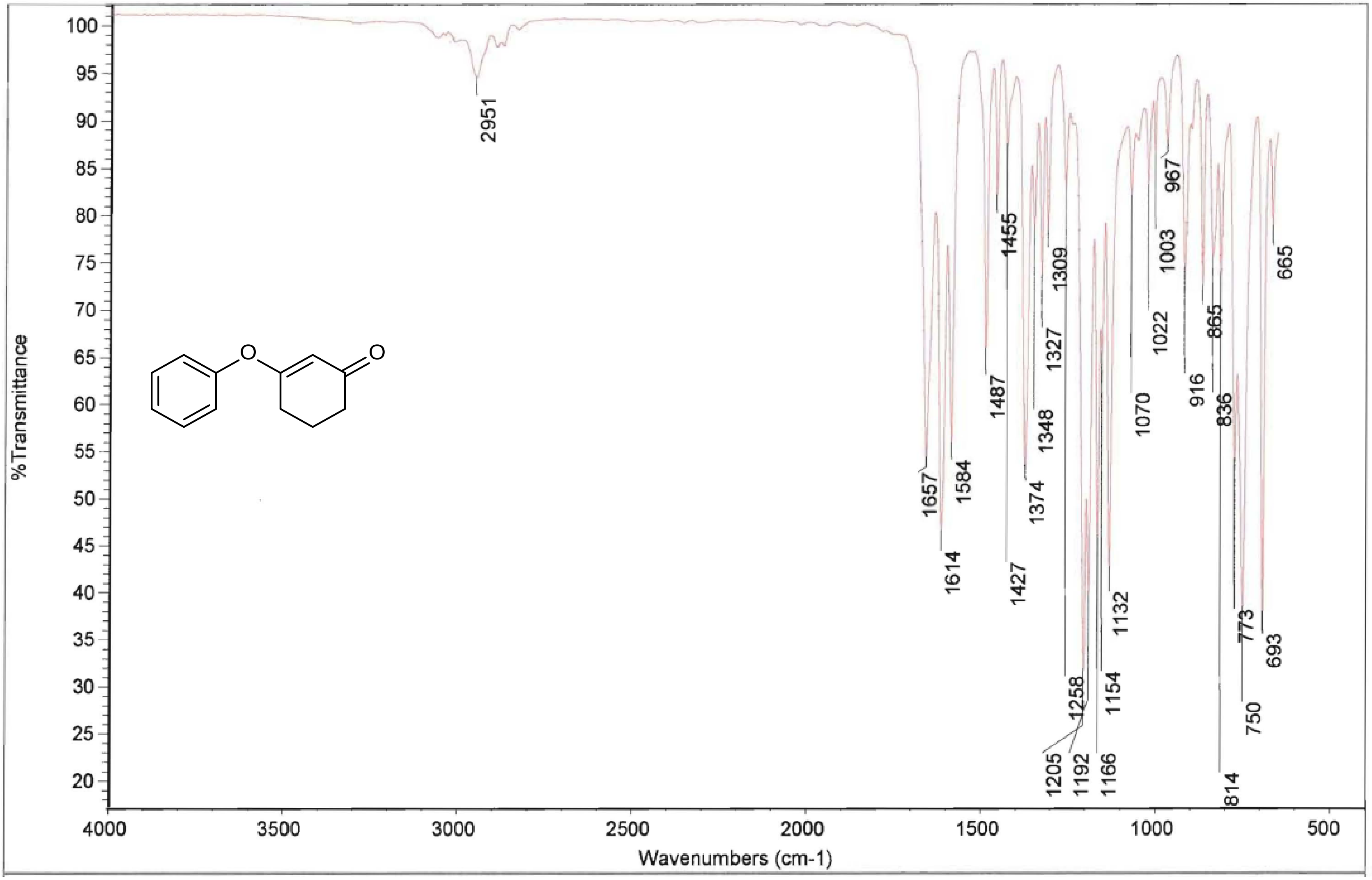

Compound 1i – ^{1}H NMR

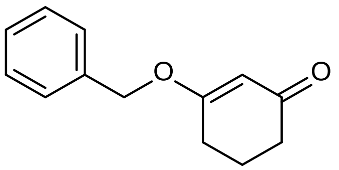

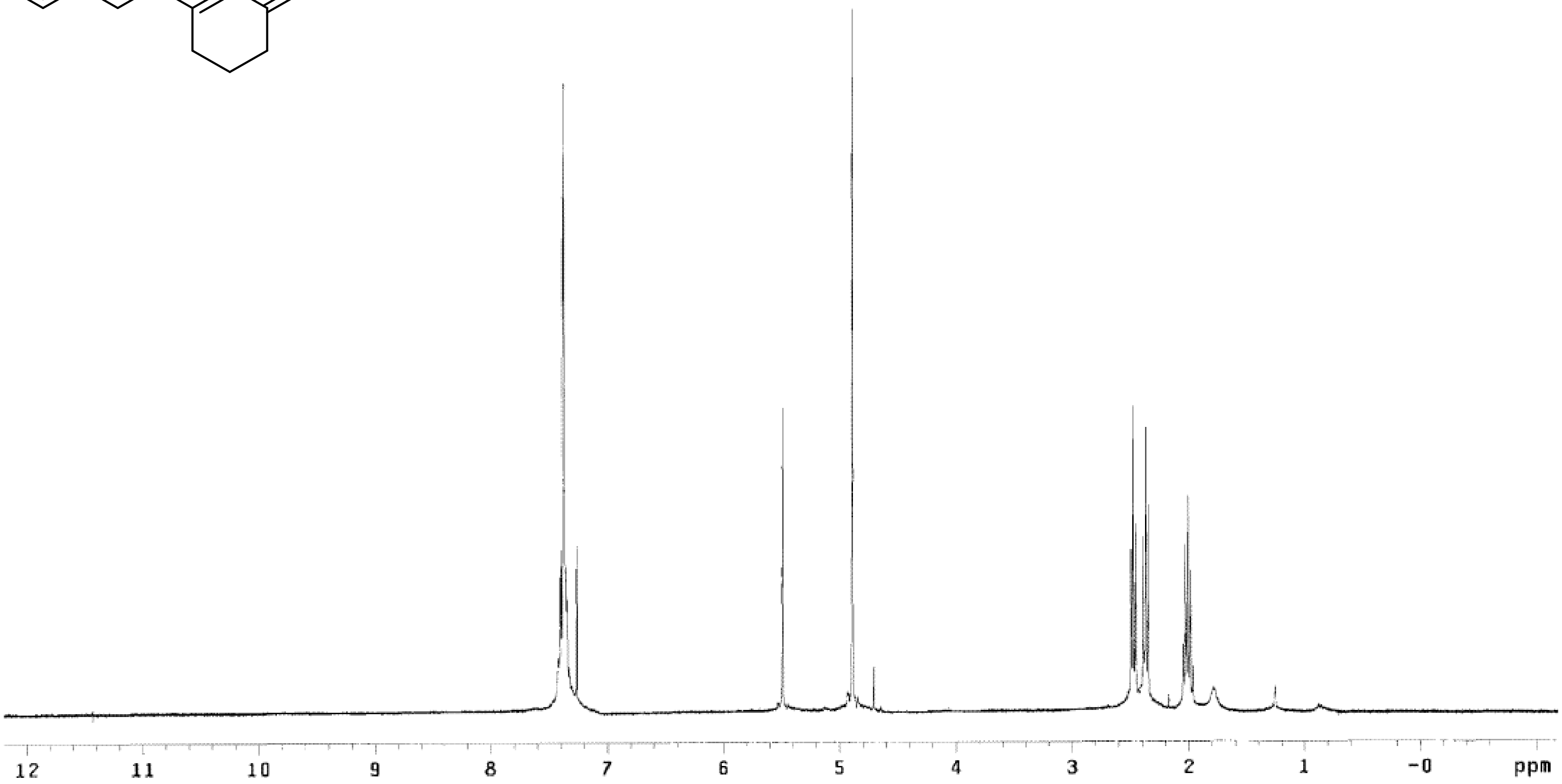

Compound 1i – ^{13}C NMR

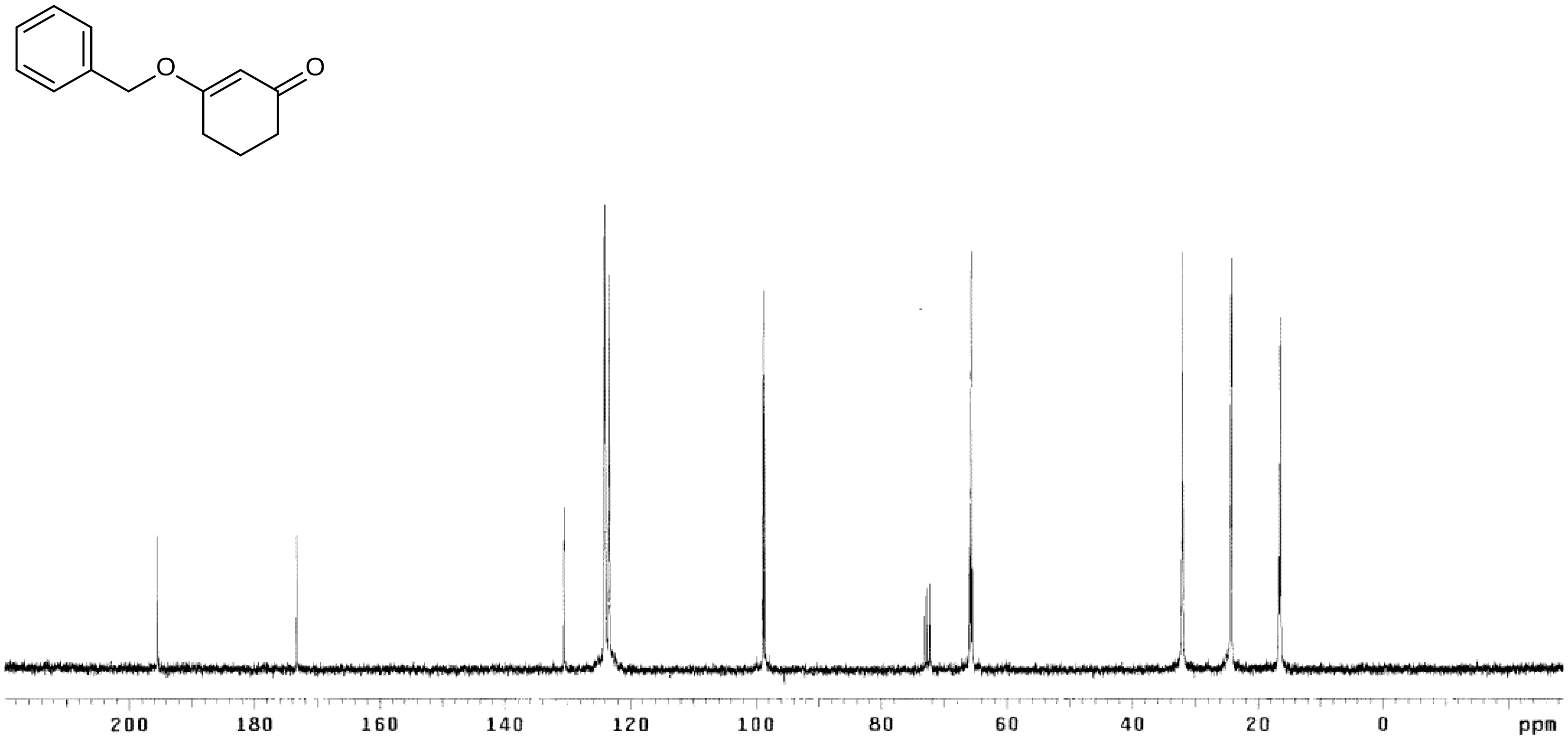

Compound 1i – IR

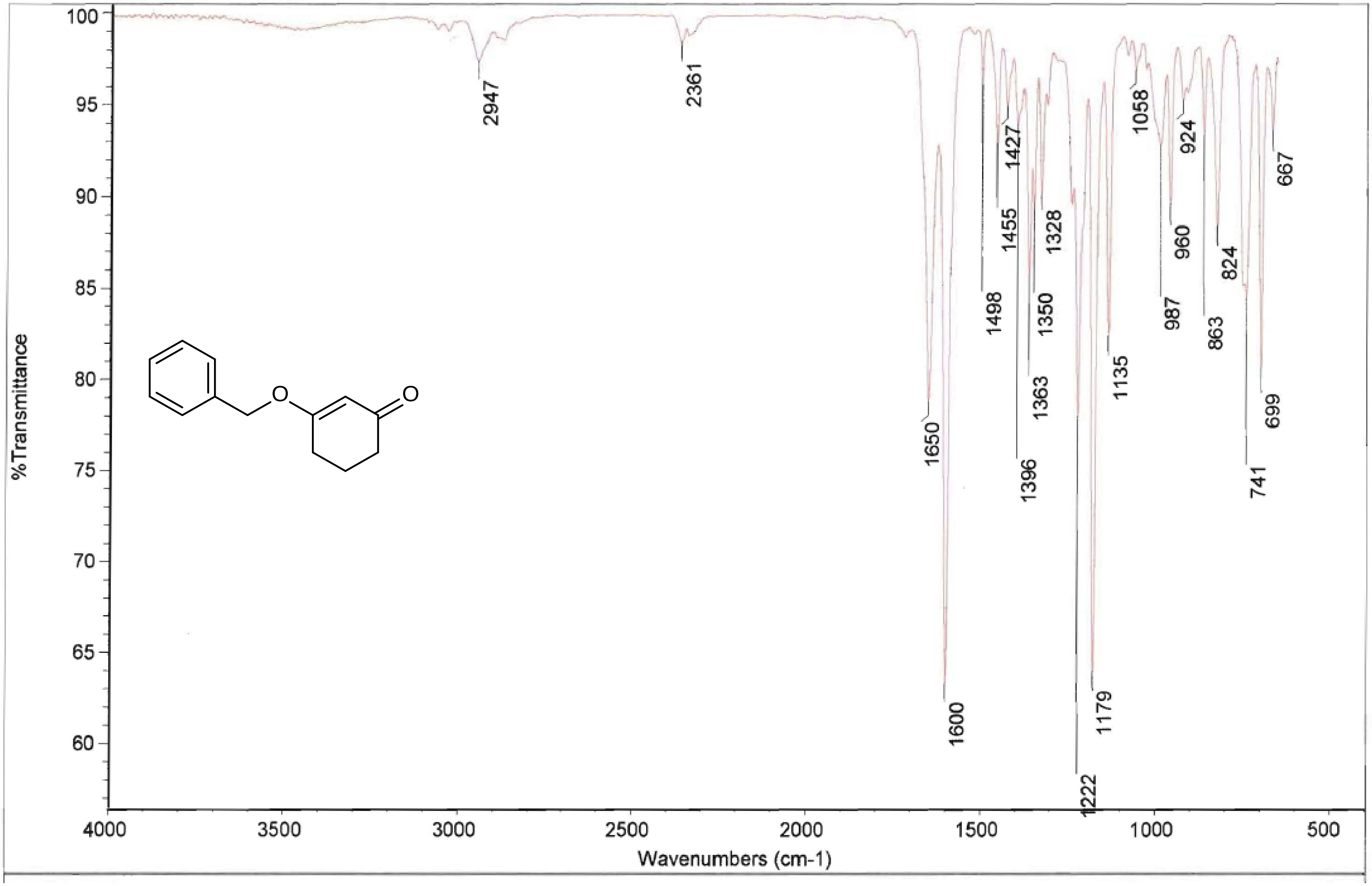

Compound 1j – ^{1}H NMR

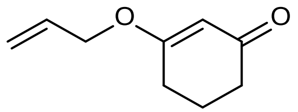

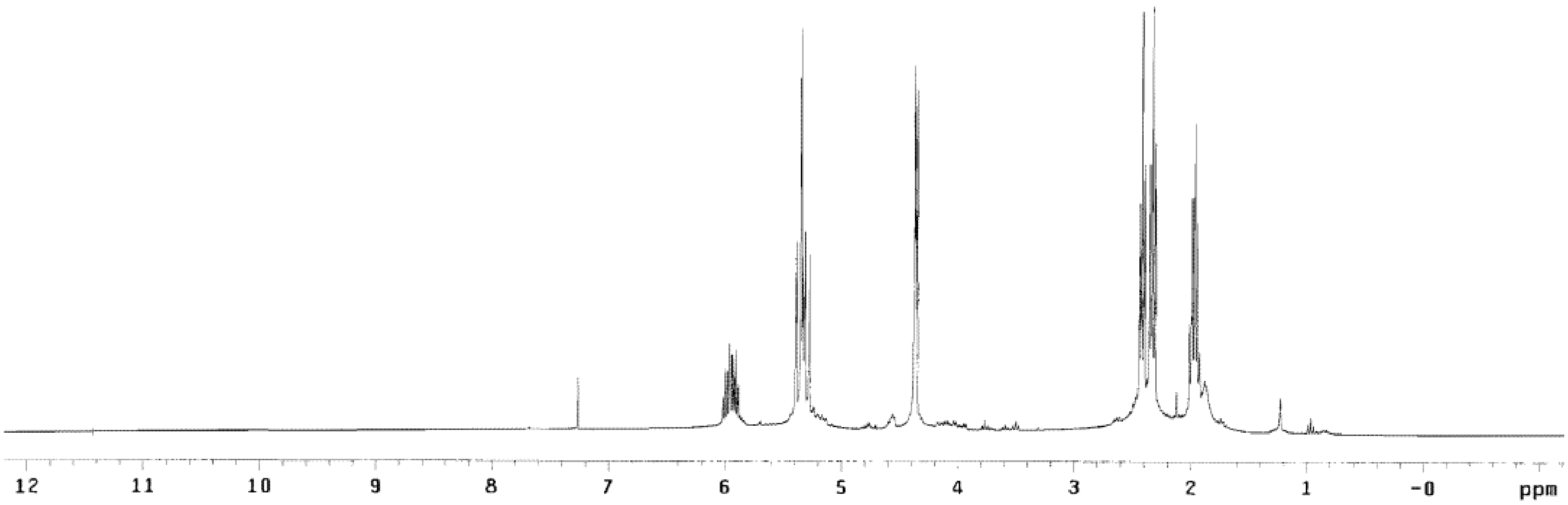

Compound 1j – ^{13}C NMR

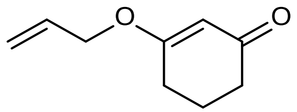

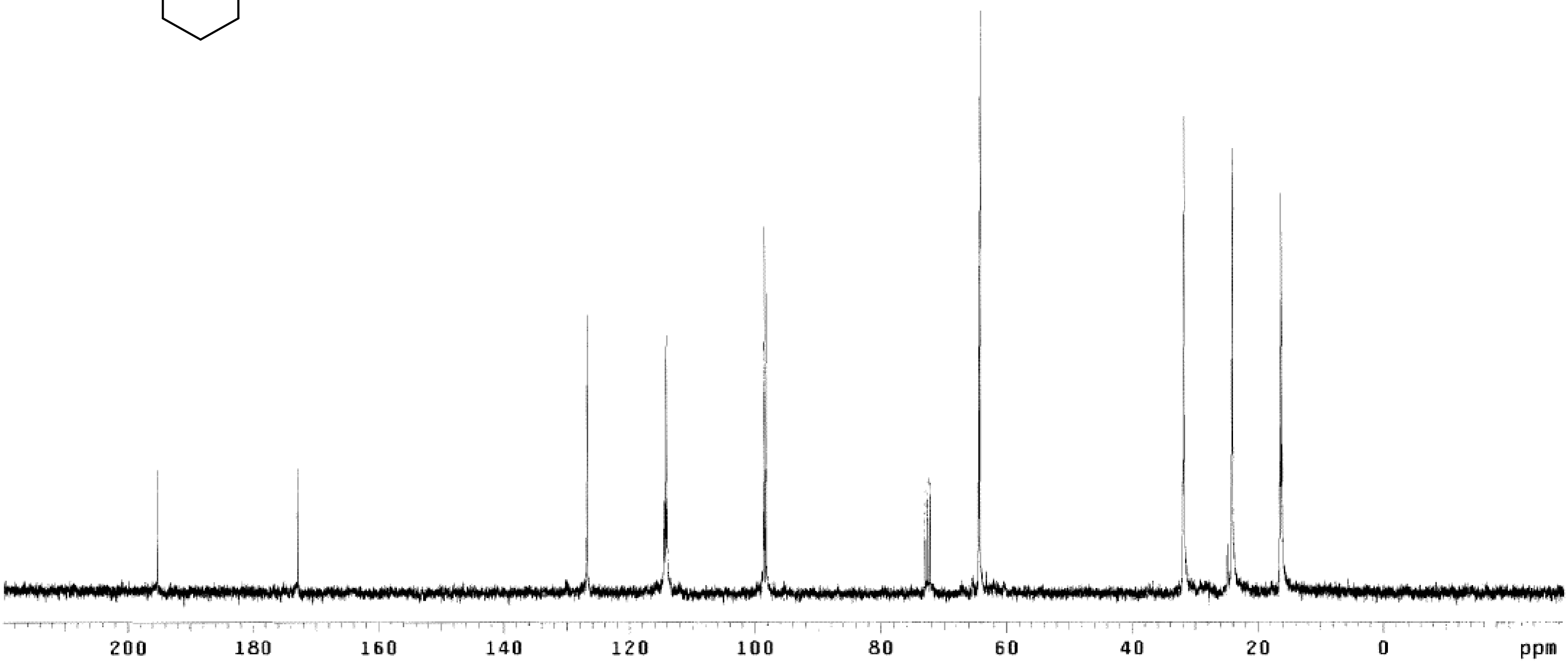

Compound 1j – IR

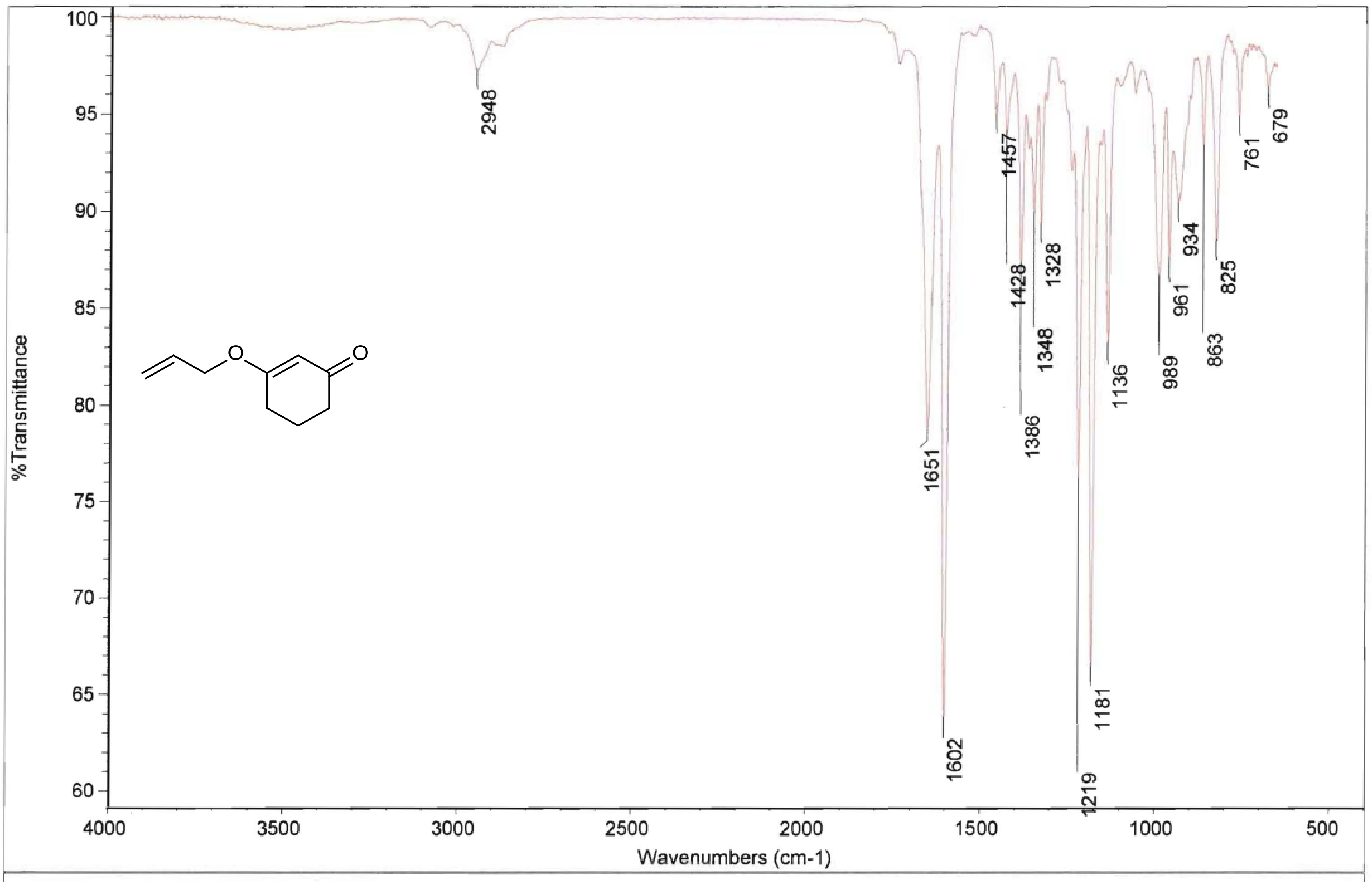

Compound 4 – ^{1}H NMR

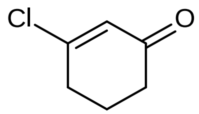

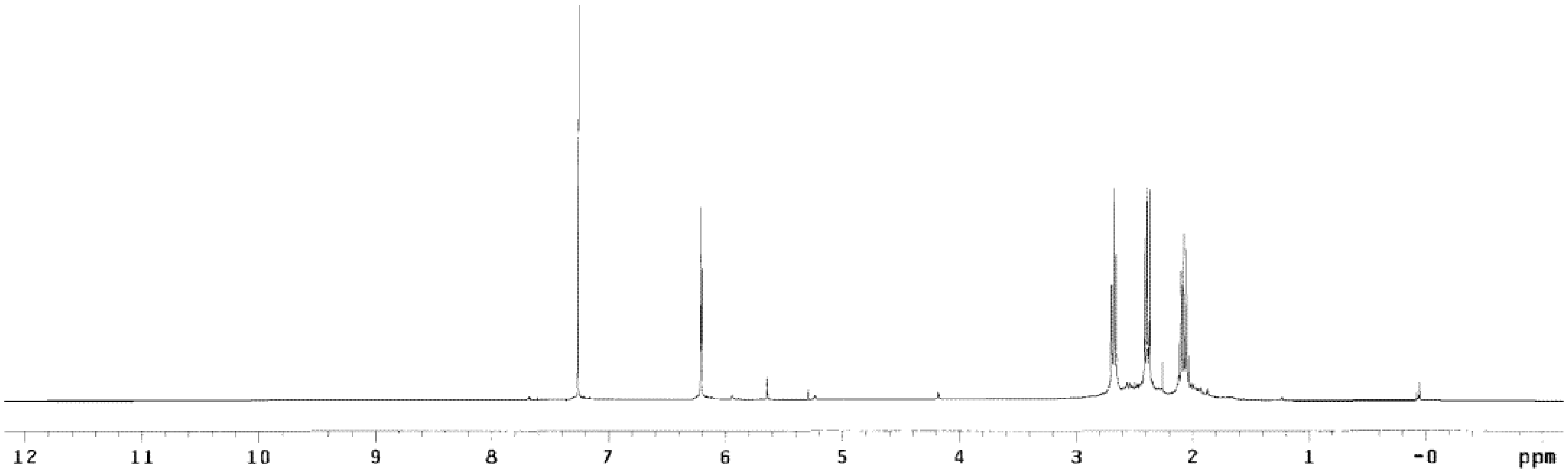

Compound 4 – ^{13}C NMR

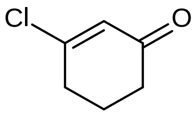

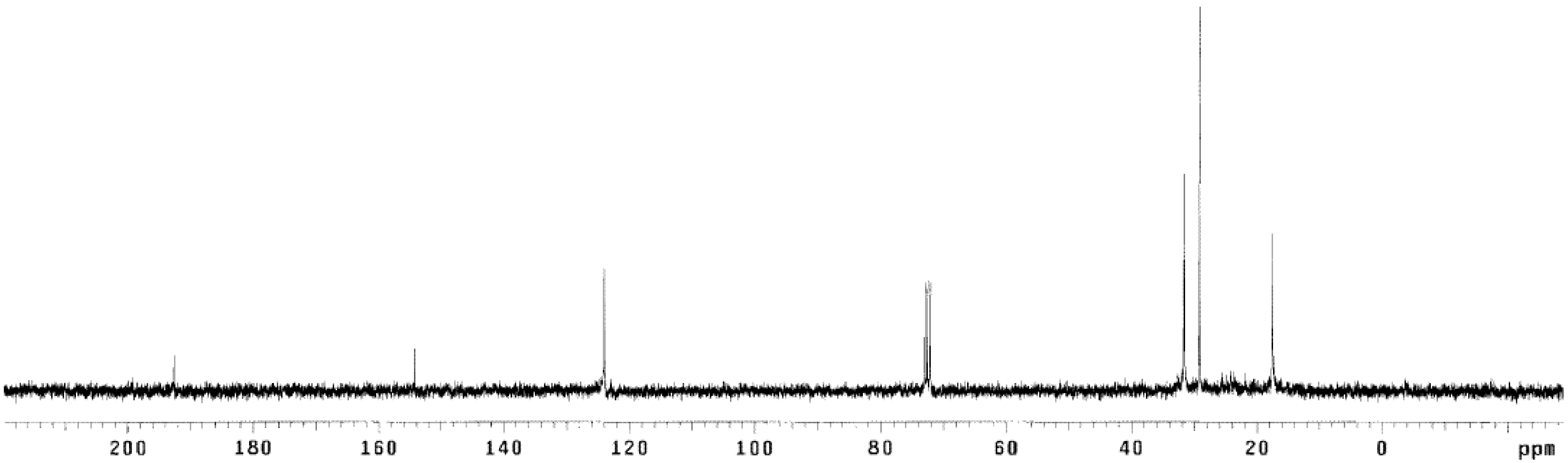

Compound 4 – IR

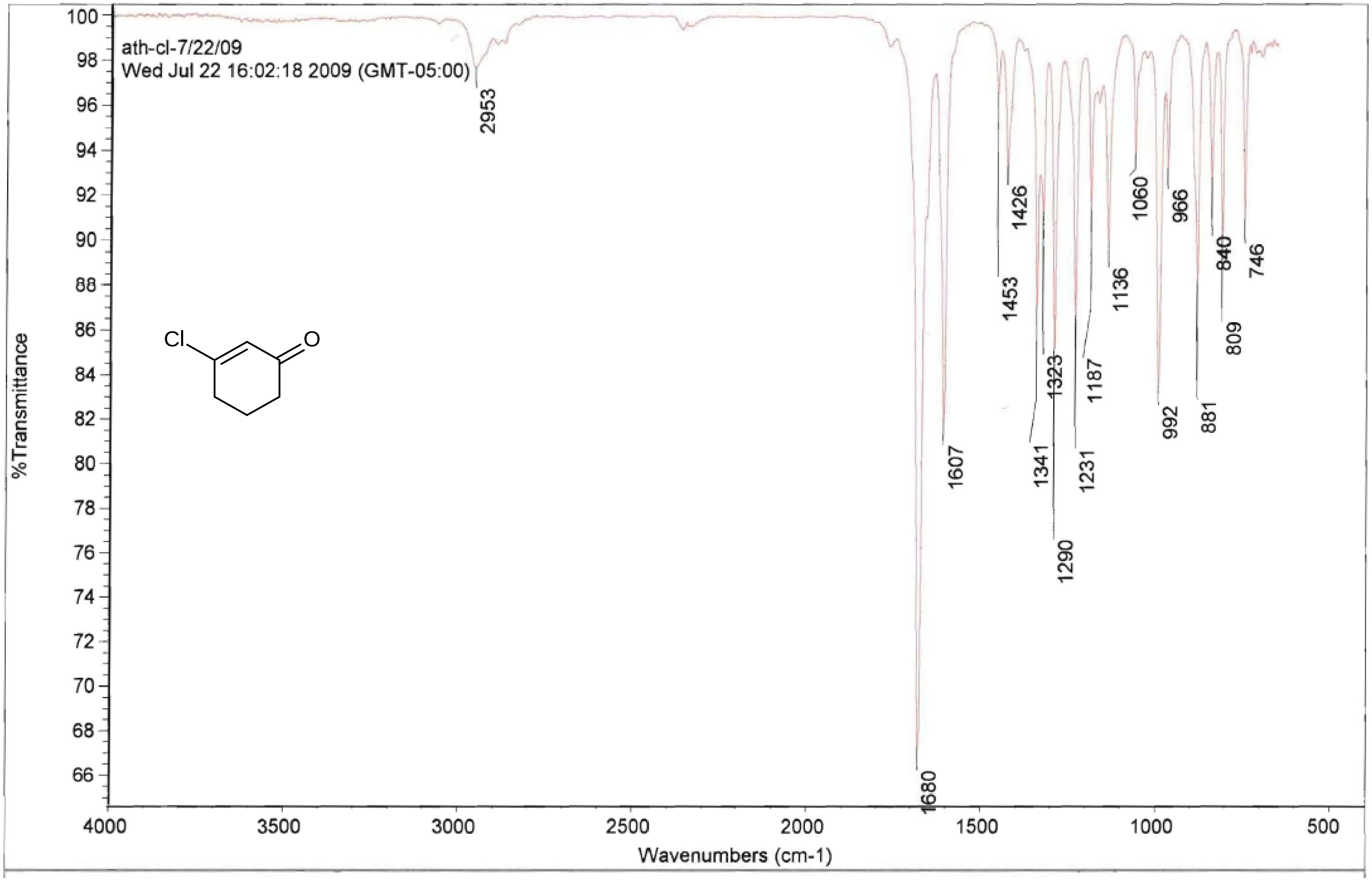

Supplement: File 1 — IR and NMR data for compounds 1a–f, 1h–j and 4. [file Beilstein_J_Org_Chem-07-1323-s001.pdf]
